# Diminished Maternal Tryptophan Leads to Sexually Dimorphic Differences in the Placenta–Brain Axis

**DOI:** 10.3390/nu18152575

**Published:** 2026-08-06

**Authors:** Rosalind T. B. Herrington, Zhen Lyu, Sarah E. Seda, Emmett E. Boling, Andrea K. Goldstein, Nathan J. Bivens, Zhentian Lei, Tanhaul Islam, Lloyd W. Sumner, Trupti Joshi, Cheryl S. Rosenfeld

**Affiliations:** 1 Pathobiology and Integrative Biomedical Sciences, University of Missouri, Columbia, MO 65211, USA; grh6mm@missouri.edu (R.T.B.H.); sest8p@missouri.edu (S.E.S.); eebqpx@missouri.edu (E.E.B.); 2 Biological Sciences, University of Missouri, Columbia, MO 65211, USA; 3 Department of Biomedical Sciences, Joan C. Edwards School of Medicine, Marshall University, Huntington, WV 25703, USA; lyuz@marshall.edu (Z.L.); joshitr@marshall.edu (T.J.); 4 Christopher S. Bond Life Science Center, University of Missouri, Columbia, MO 65211, USA; leiz@missouri.edu (Z.L.); sumnerlw@missouri.edu (L.W.S.); 5 Animal Sciences, University of Missouri, Columbia, MO 65211, USA; 6 Obstetrics, Gynecology, and Women’s Health, University of Missouri, Columbia, MO 65211, USA; agv68@health.missouri.edu; 7 Genomics Technology Core Facility, University of Missouri, Columbia, MO 65211, USA; bivensn@missouri.edu; 8 University of Missouri Metabolomics Center, University of Missouri, Columbia, MO 65211, USA; tifd3@missouri.edu; 9 Division of Biochemistry, College of Agriculture, Food and Natural Resources, University of Missouri, Columbia, MO 65211, USA; 10 Department of Electrical Engineering and Computer Science, University of Missouri, Columbia, MO 65201, USA; 11 MU Institute for Data Science and Informatics, University of Missouri, Columbia, MO 65211, USA; 12 Department of Genetics Area Program, University of Missouri, Columbia, MO 65211, USA; 13 Department of Thompson Center for Autism and Neurobehavioral Disorders, University of Missouri, Columbia, MO 65211, USA

**Keywords:** trophoblast, pregnancy, maternal diet, transcriptomics, metabolomics, serotonin, nutrition

## Abstract

**Background:** Tryptophan (Trp) is critical to mothers and their conceptuses, and this amino acid is the precursor to serotonin (5-HT). 5-HT modulates placenta function and fetal neurodevelopment. It is not clear if the placenta directly synthesizes 5-HT or uses the serotonin transporter (SERT/*Slc6a4*) to accrue 5-HT from the dam. The hypothesis tested herein is that reduction in maternal Trp leads to reductions in maternal Trp, 5-HT, and 5-hydroxy-3-indoleacetic acid (5-HIAA, metabolite of serotonin) and reductions in these metabolites within the placenta and fetal brain of female and male conceptuses. **Methods:** Female mice were placed on a reduced tryptophan (Trp) diet (0.1%) or control diet (0.2%). Diets were provided for two weeks prior to breeding (periconception period) until conceptuses were collected at approximately 12.5 days post-coitus (dpc). **Results:** While no reductions in maternal Trp and 5-HT were observed, both metabolites were significantly reduced in the placenta and fetal brain of male and female conceptuses (*p* < 0.05). Female conceptuses were susceptible to reductions in maternal Trp with 549 and 29 transcripts altered in the female placenta and fetal brain, respectively, of reduced Trp dams compared to control dams. Transcriptomic changes, such as reduced expression in *Slc6a4* and *Slc6a19* (transporter for Trp), in placenta of female conceptuses correlated with reductions in Trp and 5-HT amounts. **Conclusions:** Findings might have clinical importance to pregnant women as they reveal even subtle reductions in one amino acid profoundly influence conceptus development and might lead to sexual disparity in risk for later diseases, including neurobehavioral disorders.

## 1. Introduction

The conceptus is dependent upon the mother for provision of nutrients, including essential amino acids. As the primary communication organ with the mother, the placenta receives all nutrients and gases from underlying uterine blood vessels and returns fetal waste into maternal circulation for excretion. The placenta also relays the products received from the mother to the developing fetal organs. Amino acids are the building blocks for peptides and longer protein molecules. Placental deficiency in amino acid transport results in suppressed fetal growth [1,2]. The amino acid tryptophan (Trp) serves as the precursor for serotonin (5-HT). All Trp must be acquired through the diet with corn and sorghum providing minimal amounts of this essential amino acid [3]. Thus, in countries where such foods are the main sources of nutrition, individuals are prone to Trp deficiencies. There are other etiologies for reduced Trp levels, including Hartnup disease—a genetic condition causing reduced absorption of Trp in the gastrointestinal tract and reabsorption in the renal tubules [4]—Crohn’s disease [5], and preeclampsia [6].

In pregnant women, Trp reductions might result in harmful effects to the mother and her unborn offspring. Female mice on a completely deficient Trp diet bred to male mice show impaired decidualization response at day 5 and no implantation sites by day 8 [7]. Findings reveal that complete Trp deficient mice are unable to become pregnant. However, no differences in uterine receptivity molecules were noted between a reduced Trp group and control group of female mice [7]. While mice on the reduced Trp diet can become pregnant, concern remains as to how this might impact their developing conceptus.

5-HT is critical for normal placental and fetal development [8], thus the importance of its precursor, Trp, in pregnancy can be extrapolated and was the focus of a recent review article [9]. The article highlighted a cohort study with over 1000 female participants showing that elevation in maternal 5-hydroxytryptophan (5HTP) that might result in 5-HT deficiency is associated with small fetal size and potentially fetal growth restriction (defined as estimated fetal weight less than the tenth percentile) [10]. It also included various mouse studies. One study demonstrated that maternal imbalances of 5-HT lead to teratogenic effects in the conceptus, including congenital malformations of the central nervous system [11,12,13,14]. In fact, injection of 5-HT into pregnant mice during the pre-implantation period leads to miscarriage [11,12,13,14]. In mice, uterine inflammation due to intra-amniotic injection of bacterial lipopolysaccharide (LPS) downregulates tryptophan hydroxylase (TPH) expression in the placenta with concomitant reduction in 5-HT levels [15]. This treatment also suppresses TPH expression but increases monoamine oxidase expression in the fetal brain with a resultant decreased level of 5-HT [15].

5-HT accumulates in the human placenta [16], but the source of placental 5-HT remains unclear, as to whether it is synthesized in significant amounts by trophoblast (TB) cells or internalizes maternally produced 5-HT by binding of this compound to the serotonin transporter (SERT) expressed by trophoblast cells [17,18]. The genetic machinery for synthesis and metabolism is present in TB cells [19,20,21,22,23]. 5-HT immunoreactivity is visible in the ectoplacental cone [24,25], but during the emergence of the junctional zone (E9-12), it is localized largely in parietal trophoblast giant cells (pTGC) which are in close proximity to uterine decidual tissue [24]. While debate continues as to whether the placenta synthesizes 5-HT from maternal Trp [20,25,26,27,28] or acquires maternal 5-HT [18,24,29], the placenta provides 5-HT for the early stages of fetal forebrain development [24,26].

During early fetal brain development, 5-HT acts as both a neurotransmitter and morphogen. Some of the roles for 5-HT during this process include mediating early neural crest formation, metamorphosis, and the development of the dorsal thalamus brain region [30].

Current results reveal maternal Trp and 5-HT are essential for pregnancy, but the ramifications of reduced maternal Trp on the development of the placenta and fetal brain remain unclear. The hypothesis tested herein is that reduction in dietary Trp provided to pregnant female mice leads to reduced circulating concentrations of Trp and 5-HT in the dam and in the placenta and fetus of both female and male conceptuses. Secondarily, we postulated that reduced maternal Trp might cause global transcriptomic changes in the placenta and fetal brain in female and male offspring. To test these hypotheses, C57 female mice were fed a reduced tryptophan diet (0.1% Trp) or control diet (0.2% Trp). These diets were administered for two weeks prior to breeding until euthanasia at ~12.5 days post-coitus (dpc). At this time, maternal serum and uterine samples were collected to analyze amounts of Trp, 5-HT, and 5-HIAA (metabolite of 5-HT) in these samples. These metabolites were also measured in placenta and fetal brain samples from female and male conceptuses. RNA was also isolated from these tissues and used for RNAseq analyses to assess sex-dependent transcriptomic changes in these organs.

## 2. Materials and Methods

### 2.1. Placement of Female Mice on Reduced Tryptophan and Control Tryptophan Diet and Breeding

This research was approved by the University of Missouri Animal Care and Use Committee (ACUC, Protocol #45687). Twelve adult (~8–12 week old) wild type C57Bl6J mouse dams, which were purchased from Jackson Laboratory (Bar Harbor, ME, USA), were randomly assigned to be placed on one of two diets: (1) reduced in Trp (0.1%, Trp, 190563, Table S1) or (2) control amount of tryptophan-Trp (0.2%, 190359, Table S2). The diets were formulated by Inotiv (previously Teklad, Madison, WI, USA). These diets and percentages have been chosen based on previous publications [7,31]. Yusufu et al. [31] showed that the reduced Trp diet tested herein results in gut microbiome and other phenotypic changes. A maternal diet completely deficient in Trp resulted in no implantation sites [7], whereas no differences in uterine receptivity were noted in the reduced Trp vs. CTL Trp diet [31]. However, these researchers did not examine how these diets might impact maternal behavior, including sleeping, and the placenta–brain axis in her fetuses. Notably, neither of these studies reported any gross adverse changes due to consumption of the reduced Trp diet. The mice consumed both diets to the same extent. We closely monitored diet intake and health of the females on both diets, and no adverse effects were noted.

After the females were randomly assigned to one of the two diets, we analyzed the average and standard error of the mean (SEM) weight to ensure the two groups did not vary in weight at the outset of the studies. Mice were housed in a 12:12 h dark: light cycle. Aspen shaving bedding from Bourn Feed (Columbia, MO, USA) was provided. Each female was given a nestlet (Ancare Corp., Bellmore, NY, USA) for environmental enrichment and nest building. Cages, aspen bedding, and nestlets were changed weekly as required by our animal facility. Cages for the two treatment groups were randomized for each shelf and rack to avoid any positional bias. Females were housed singly to monitor food intake. We and others who have analyzed how maternal changes in mice can affect the placenta have primarily tested four to six replicates per group [32,33,34,35,36,37,38,39,40], which is based on a power analysis of 0.8. Females were fed one of these two diets for two weeks prior to breeding (periconception period). Females were paired each night to control C57Bl6J males and examined the next morning for a vaginal plug. Males were removed at this time. If no vaginal plug was observed, the male was re-introduced that night, and female plugs were checked again the next morning until all had been bred. Pregnant females were maintained on one of the two assigned diets until euthanasia at ~12.5 dpc.

### 2.2. Collection of Maternal Serum, Maternal Uterine Samples, and Fetal Placenta and Brain Samples from Pregnant Mice on Reduced Tryptophan and Control Tryptophan Diets

Females from both groups who had been previously bred, as detailed above, were weighed daily and humanely euthanized at ~12.5 dpc, and this was done in accordance with American Veterinary Medical Association (AVMA)-approved euthanasia methods (CO_2_ followed by cervical dislocation). Fetal placentas and brains were frozen in liquid nitrogen. Fetal tissue was collected for embryonic sexing procedures. We, and others, have shown that this period is when the placenta is vulnerable to in utero perturbations [32,33,34,35,36,37,38,39]. Additionally, the brain is initially formed around this time and responsive to placental-derived factors, in particular 5-HT [41,42]. The experimental unit for the current work was 4-6 F1 male and female placenta or brain samples from conceptuses derived from P0 females on either the Trp reduced or Trp control diet. The Trp reduced and Trp control diet had 6 females in each case. All the females became pregnant, resulting in 6 litters for each group. Based on the PCR analysis to determine sex of the fetus (detailed below), and Fragment Analyzer results (detailed below), one female conceptus and one male conceptus from each litter was analyzed for metabolomics and RNAseq studies. This approach was used to avoid pseudo-replication by sampling more than one conceptus from each litter. The reason some F1 placenta or fetal brain samples only had four replicates is that for a few samples insufficient RNA was isolated for the RNAseq studies. The total animals included in the study for the P0 females and F1 male and female conceptuses was 36.

### 2.3. PCR Sexing of Conceptuses from Germ-Free and Multi-Pathogen-Free Pregnant Mice

Sex analysis of embryonic tissues was performed based on the presence or absence of an *Sry* gene, as we have done previously [43,44,45]. To ensure the absence of a band was not due to inadequate quality of DNA, primers against *Gapdh* were simultaneously analyzed and run on a separate gel.

### 2.4. Metabolomics Analyses of Tryptophan (Trp), Serotonin (5-HT), and 5-Hydroxy-3-Indoleacetic Acid (5-HIAA) in Maternal Serum and Uterine Samples and Placenta and Fetal Brain Samples from Conceptuses

Uterine, placenta, and fetal brain samples were weighed and diluted to a ratio of 1:20 (*w*/*v*; mg/µL) in acetonitrile containing 0.1% formic acid and a stable isotope-labeled internal standard mixture consisting of tryptophan-d5, serotonin-d4, and 5-hydroxy-3-indoleacetic acid-d6 (1.5 μg/mL each). The diluted tissue samples were homogenized by using a bead beater, transferred to Eppendorf tubes, and centrifuged at 13,000× *g* for 15 min. Maternal serum samples (50 μL) were extracted with 950 µL of the same solvent system containing the internal standards. The serum samples were vortexed and shaken on an orbital shaker for 30 min, followed by centrifugation at 13,000× *g* for 15 min. After centrifugation, 250 µL of the supernatant from each sample (uterine tissue, placenta, fetal brain, and maternal serum) was transferred to glass vials and evaporated to dryness under a gentle stream of nitrogen. The dried extracts were reconstituted in 75 μL of methanol containing 0.01% formic acid, yielding a final internal standard concentration of 5 μg/mL for each labeled compound. The reconstituted samples were transferred to glass inserts for subsequent liquid chromatography mass spectrometry (LC–MS) analysis.

LC–MS/MS analysis was performed in multiple reaction monitoring (MRM) mode by using a Waters Xevo TQ XS (Waters Corp., Milford, MA, USA) coupled to a Waters UHPLC system. Ionization was carried out with a positive electrospray ionization mode (ESI+). Chromatographic separation was achieved by using a C18 column (2.7 μm, 2.1 × 150 mm). The mobile phases consisted of (A) water with 0.1% formic acid and (B) acetonitrile. The gradient elution program (%B) was as follows: 0–0.15 min, 2%; 0.15–10.50 min, 2–60%; 10.50–11.00 min, 60–99%; held at 99% until 13.00 min; 13.00–13.50 min, 99–2%; and re-equilibrated at 2% until 16.00 min. The flow rate was set to 0.3 mL/minute. The column temperature was maintained at 40 °C, and the autosampler temperature was set to 10 °C. Mass spectrometric conditions were as follows: desolvation temperature, 250 °C; desolvation gas flow, 600 L/h; and cone gas flow, 150 L/h. The transition in MRM mode is shown below:

External calibration curves were generated for tryptophan, serotonin, and 5-hydroxy-3-indoleacetic acid by using a standard dilution series prepared in the presence of the isotope-labeled internal standards (5 μg/mL each). Quantification was performed based on the analyte-to-internal standard peak area ratios. Multivariate statistical analysis was conducted with MetaboAnalyst (Version 6.0). Principal component analysis (PCA) was applied to visualize sample clustering and metabolic variation. Data were analyzed with t-test analysis that was done with Microsoft Excel. A *p* value ≤ 0.05 was considered statistically significant.

### 2.5. RNA Isolation from Fetal Placenta and Brain Samples

To characterize changes in gene expression patterns in placenta and fetal brain samples from female and male conceptuses derived from dams on the reduced Trp and control tryptophan diet, total RNA was extracted as reported previously [43,44,45]. RNA was extracted and analyzed from 6 female placentas derived from dams on the reduced Trp diet, 4 female placentas derived from dams on the control Trp diet, 5 male placentas derived from dams on the reduced Trp diet, 6 male placentas derived from dams on the control Trp diet, 6 female fetal brain samples derived from dams on the reduced Trp diet, 4 female fetal brain samples derived from dams on the control Trp diet, 4 male fetal brain samples derived from dams on the reduced Trp diet, and 6 male fetal brain samples derived from dams on the control Trp diet. For each litter, we selected one female and one male conceptus for these studies. RNA integrity and quantity was determined by using the Fragment Analyzer system (University of Missouri Genomics Technology Core, Columbia, MO, USA). Only those samples with an RNA integrity number (RIN) score ≥ 7.0 were used for RNA sequencing (RNAseq).

### 2.6. Illumina TruSeq RNA Library Preparation and Sequencing

Libraries were constructed following the manufacturer’s protocol with reagents supplied in the Illumina mRNA Stranded Library Preparation Kit (Illumina Inc., San Diego, CA, USA) and sequenced at the University of Missouri Genomics Technology Core as described previously [43,44,45].

### 2.7. RNA-seq Data Processing

The RNA-seq data analysis pipeline commenced with quality assessment of the raw Illumina sequencing reads using FastQC (version 0.12.1) [46]. High-quality reads were subsequently aligned to the mouse reference genome (GRCm39) by using the HISAT2 (version 2.1.0) aligner [47]. Gene-level read counts were then quantified with the featureCounts program (version 1.42.1) [48]. The DESeq2 package (version 1.42.1) in R was used to determine differentially expressed genes (DEGs) based on sex and fetal tissue and was based on an adjusted *p*-value (padj) ≤ 0.05 and an absolute log_2_ fold change (|log2FC|) > 1 [49].

Principal Component Analysis (PCA) was conducted on normalized gene expression counts based on the scikit-learn library (version 1.6.1) [50]. Volcano plots were generated for each comparison by using the matplotlib package (version 3.10.0) [51]. This analysis was done to visualize significantly upregulated and downregulated genes for each comparison.

### 2.8. Enrichment and Network Analyses

Enrichment analyses were determined based on the free-only program, TissueEnrich [52] that uses the mouse ENCODE dataset [53] and EnrichR (version 3.4) [54] with the KEGG_2019_Mouse database. DEGs were imported into G:Profiler to analyze GO pathways, including molecular function, biological function, and cellular component, that are predicted to be affected based on the transcriptomic changes identified in the female placenta and fetal brain for those conceptuses derived from dams on the reduced Trp diet compared to those derived from dams on the control Trp diett [55].

### 2.9. Correlation Analyses of RNAseq and Metabolomics Data

DEGs identified from each comparison were correlated with metabolomics data by using R. Correlation analyses were determined based on Kendall’s rank correlation coefficient, and this procedure was done to assess monotonic associations between gene expression levels and metabolite abundances. Results were visualized with the corrplot package (version 0.95) [56]. Statistically significant correlations (*p* < 0.05) are highlighted with red rectangles in the figures to emphasize meaningful gene–metabolite associations.

## 3. Results

### 3.1. Maternal Body Weight, Food Consumption, and Litter Size

No differences in body weight were noted prior to being placed on either the control Trp diet or reduced Trp diet or at the time of euthanasia (*p* > 0.05 for both timepoints, Figure S1A). No difference in average food consumption was observed between the two groups (reduced Trp diet: 0.74 ± 0.02 g and control Trp diet: 0.72 ± 0.03 g, *p* = 0.59). No differences in litter size were noted between pregnant female mice on the reduced Trp diet compared to those on the control Trp diet (8.17 ± 0.64 vs. 8 ± 1.13 conceptuses, respectively, *p* = 0.90, Figure S1B).

### 3.2. Tryptophan (Trp), Serotonin (5-HT), and 5-HIAA Concentrations Within Maternal Serum and Uterine Samples

No significant differences in Trp, 5-HT, or 5-HIAA were detected in maternal serum and uterine samples. These results are provided in Table S4. Trp in serum from reduced Trp dams and control Trp dams was 6.96 ± 2.83 ng/mL vs. 13.05 ± 2.45 ng/mL, respectively, *p* = 0.07. 5-HT in serum from reduced Trp dams and control Trp dams was 0.803 ± 0.59 vs. 1.62 ± 0.75, respectively, *p* = 0.23. 5-HIAA in serum from reduced Trp dams and control Trp dams was 0.106 ± 0.08 vs. 0.077 ± 0.02, respectively, *p* = 0.35. Trp in uterine tissue from reduced Trp dams and control Trp dams was 9.45 ± 2.96 vs. 8.26 ± 1.81, respectively, *p* = 0.37. 5-HT in uterine tissue from reduced Trp dams and control Trp dams was 0.001 ± 0.0005 vs. 0.001 ± 0.0004, respectively, *p* = 0.40. 5-HIAA in uterine tissue from reduced Trp dams and control Trp dams was 0.24 ± 0.07 vs. 0.22 ± 0.05, respectively, *p* = 0.42.

### 3.3. Tryptophan (Trp), Serotonin (5-HT), and 5-HIAA Concentrations Within Placenta and Fetal Brain Samples

**Figure 1:**
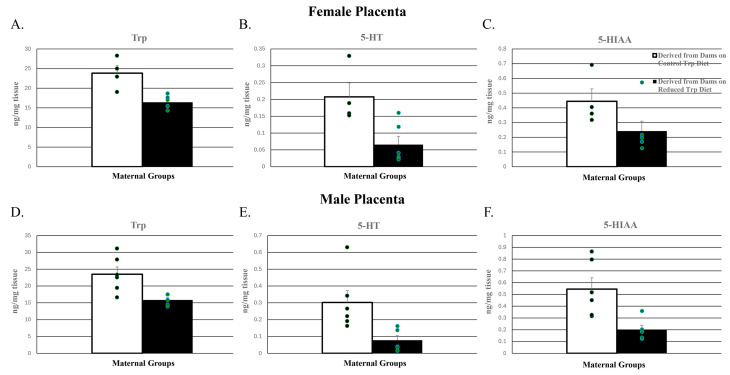
Trp, 5-HT, and 5-HIAA concentrations in female and male placenta samples. (**A**) Comparison of Trp concentrations (ng/mg tissue) in female placentas derived from conceptuses of control Trp dams vs. female placentas derived from conceptuses of control Trp dams; *p* = 0.001. (**B**) Comparison of 5-HT concentrations (ng/mg tissue) in female placentas derived from conceptuses of control Trp dams vs. female placentas derived from conceptuses of control Trp dams; *p* = 0.007. (**C**) Comparison of 5-HIAA concentrations (ng/mg tissue) in female placentas derived from conceptuses of control Trp dams vs. female placentas derived from conceptuses of control Trp dams; *p* ≤ 0.05. (**D**) Comparison of Trp concentrations (ng/mg tissue) in male placentas derived from conceptuses of control Trp dams vs. male placentas derived from conceptuses of control Trp dams; *p* = 0.007. (**E**) Comparison of 5-HT concentrations (ng/mg tissue) in male placentas derived from conceptuses of control Trp dams vs. male placentas derived from conceptuses of control Trp dams; *p* = 0.01. (**F**) Comparison of 5-HIAA concentrations (ng/mg tissue) in male placentas derived from conceptuses of control Trp dams vs. male placentas derived from conceptuses of control Trp dams; *p* = 0.007.

While no differences were detected for these three molecules in maternal serum and uterine tissue, striking differences were detected in the placenta and fetal brain from female and male conceptuses. Female placentas from pregnant mice on a reduced Trp diet had significantly reduced concentrations of Trp (16.43 ± 0.66 vs. 23.8 ± 1.93, respectively, *p* = 0.001, Figure 1A, Table S5). 5-HT was also reduced in the placentas of female conceptuses derived from dams on the reduced Trp diet compared to those born to control dams (0.07 ± 0.02 vs. 0.21 ± 0.04, respectively, *p* = 0.007, Figure 1B, Table S5). Additionally, 5-HIAA was significantly reduced in the same cohort (0.24 ± 0.07 vs. 0.44 ± 0.08, respectively, *p* ≤ 0.05, Figure 1C, Table S5). Concentrations of Trp in male placentas from dams on a reduced Trp diet vs. control dams were also significantly reduced (15.86 ± 0.68 vs. 23.5 ± 2.17, respectively, *p* = 0.007, Figure 1D, Table S5). Placental samples of male conceptuses from dams on the reduced Trp diet had reduced 5-HT concentrations relative to counterparts originating from dams on the control diet (0.08 ± 0.03 vs. 0.30 ± 0.07, respectively, *p* = 0.01, Figure 1E, Table S5), as well as reduced 5-HIAA in the same cohort (0.20 ± 0.04 vs. 0.54 ± 0.10, respectively, *p* = 0.007, Figure 1F, Table S5).

**Figure 2:**
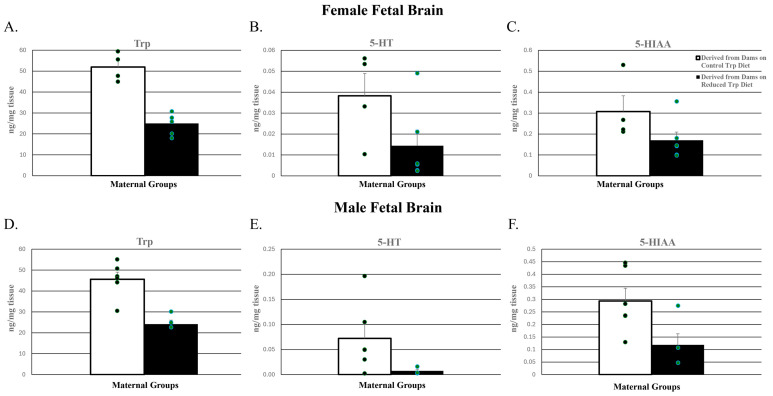
Trp, 5-HT, and 5-HIAA concentrations in female and male fetal brain samples. (**A**) Comparison of Trp concentrations (ng/mg tissue) in female fetal brains derived from conceptuses of control Trp dams vs. female fetal brains conceptuses of control Trp dams; *p* = 0.00005. (**B**) Comparison of 5-HT concentrations (ng/mg tissue) in female fetal brains derived from conceptuses of control Trp dams vs. female fetal brains derived from conceptuses of control Trp dams; *p* ≤ 0.05. (**C**) Comparison of 5-HIAA concentrations (ng/mg tissue) in female fetal brains derived from conceptuses of control Trp dams vs. female fetal brains derived from conceptuses of control Trp dams; *p* = 0.06. (**D**) Comparison of Trp concentrations (ng/mg tissue) in male fetal brains derived from conceptuses of control Trp dams vs. male fetal brains derived from conceptuses of control Trp dams; *p* = 0.0003. (**E**) Comparison of 5-HT concentrations (ng/mg tissue) in male fetal brains derived from conceptuses of control Trp dams vs. male fetal brains derived from conceptuses of control Trp dams; *p* = 0.08. (**F**) Comparison of 5-HIAA concentrations (ng/mg tissue) in male fetal brains derived from conceptuses of control Trp dams vs. male fetal brains derived from conceptuses of control Trp dams; *p* = 0.02.

Trp showed marked reduction in fetal brain samples from female conceptuses derived from dams on the reduced Trp diet relative to female fetal brain samples from control dams (25.04 ± 2.01 vs. 51.94 ± 3.37, respectively, *p* = 0.00005, Figure 2A, Table S5). 5-HT levels were reduced in female fetal brain samples vs. female brain samples from control dams (0.01 ± 0.007 vs. 0.04 ± 0.01, respectively, *p* ≤ 0.05, Figure 2B, Table S5). Lower levels of 5-HIAA were seen in fetal brain samples of female conceptuses after in utero exposure to the reduced Trp diet compared to analogous samples from control dams (0.17 ± 0.04 vs. 0.31 ±0.07, respectively, *p* = 0.06, Figure 2C, Table S5). Fetal brains from male conceptuses derived from dams on the reduced Trp diet also had significantly decreased amounts of Trp relative to those derived from control dams (24.2 ± 1.90 vs. 45.6 ± 3.41, respectively, *p* = 0.0003, Figure 2D, Table S5). Male fetal brain samples from dams on the reduced Trp diet also showed a trend towards a reduction in 5-HT compared to those from control dams (0.0075 ± 0.003 vs. 0.072 ± 0.03, respectively, *p* = 0.08, Figure 2E, Table S5). 5-HIAA amounts were significantly reduced in male fetal brain samples from dams on the reduced Trp diet relative to control dams (0.12 ± 0.04 vs. 0.29 ± 0.05, respectively, *p* = 0.02, Figure 2F, Table S5). Comparing levels of Trp in maternal and fetal tissues, Trp was present in highest concentrations in both the male and female fetal brain samples, followed by the male and female placentas, and, finally, the uterine tissue. However, 5-HT was most abundant in uterine tissue, followed by male and female placentas, and, finally, male and female fetal brain samples.

### 3.4. RNA Sequencing

#### 3.4.1. General Features Based on Organ, Genotype, and Sex Differences

The average number of reads for all samples was 131,000, the average number of mapped reads was 128,000,000, and the average percentage of alignment was 95.2% (Table S3). PCA plots for the following comparisons is provided in Figure S2: female placentas from reduced dams vs. female placentas from control Trp dams, male placentas from reduced Trp dams vs. male placentas from control Trp dams, female fetal brains from reduced Trp dams vs. female fetal brains from control Trp dams, and male fetal brains from reduced Trp dams vs. male fetal brains from control Trp dams. PERMANOVA values for female and male placental comparisons were 0.05 and 0.23, respectively. PERMANOVA values for female and male fetal brain comparisons were 0.04 and 0.26, respectively. Thus, there was significant separation for the female group comparisons but not for the male group comparisons. Importantly, these data provide the first hints that transcriptomic profiles of the placenta and fetal brain of female conceptuses are vulnerable to even slight reductions in maternal Trp dietary ingestion, whereas this change in maternal nutrient status had only minimal effect in the placenta and fetal brain of male conceptuses.

**Figure 3:**
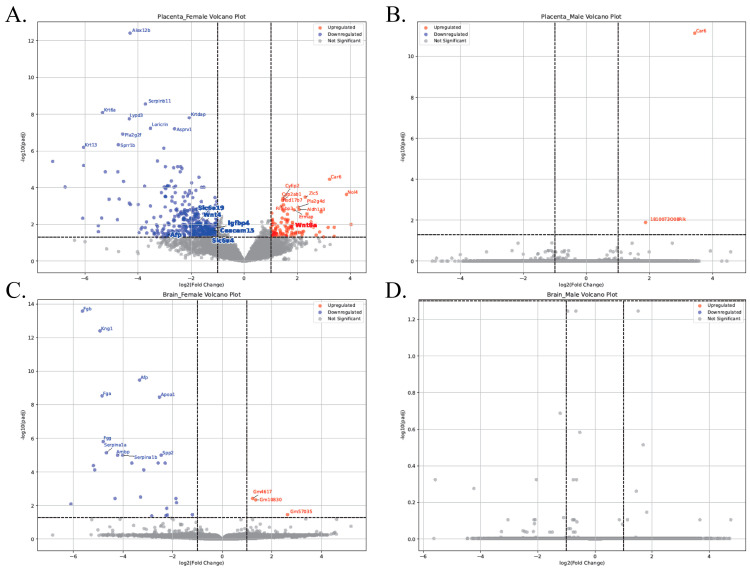
Volcano plots showing differentially expressed genes (DEGs) for female and male placentas and fetal brains from reduced Trp and control dams. (**A**) Volcano plot comparison of DEGs in female placentas derived from reduced Trp dams vs. female placentas derived from control Trp dams. (**B**) Volcano plot comparison of DEGs in male placentas derived from reduced Trp vs. male placenta derived from control Trp dams. (**C**) Volcano plot comparison of DEGs in female fetal brain samples derived from reduced Trp dams vs. female fetal brain samples derived from control Trp dams. (**D**) Volcano plot comparison of DEGs in male fetal brain samples derived from reduced Trp dams vs. male fetal brain samples derived from control Trp dams.

Volcano plot analyses revealed that at padj ≤ 0.05 and two-fold change difference, 549 transcripts differed between female placentas from reduced Trp dams vs. female placentas from control Trp dams, with 99 upregulated and 450 downregulated in female placentas from reduced Trp dams relative to female placentas from control Trp Dams (Figure 3A). For male placenta samples, two differentially expressed genes were identified between conceptuses derived from reduced Trp dams vs. control Trp dams, and both were upregulated in male placentas from reduced Trp dams relative to control Trp dams (Figure 3B). For brain samples, 29 transcripts were differentially expressed for female brain samples with three upregulated and 26 downregulated in the fetal brains of female conceptuses derived from reduced Trp dams compared to the fetal brains of female conceptuses derived from control Trp dams (Figure 3C). For male brain samples, zero genes showed differential expression between conceptuses derived from reduced Trp females vs. those from control Trp females (Figure 3D). Thus, the volcano plot analyses further emphasize how reductions in maternal Trp dietary intake lead to pronounced sex differences in gene expression alterations in the placentas and fetal brains of female conceptuses but only minimal changes for male conceptuses.

#### 3.4.2. Differentially Expressed Genes in Placenta Based on Maternal Trp Dietary Status and Conceptus Sex Differences

Of the 549 known differentially expressed transcripts in female placentas compared to the two known differentially expressed genes in male placentas, both genes differentially expressed in the male (*Car6* and 1810073O08Rik) overlapped when comparing reduced Trp vs. control Trp placental samples (Figure S3, Supplementary File S1). A list of genes that are solely differentially expressed in female placentas from reduced Trp vs. control Trp dams appears in column A, and a list of differentially expressed genes that overlap in female and male comparisons appears in column B. This Venn diagram comparison further illustrates the fact that the reduced maternal Trp diet caused dramatic gene expression changes in the placentas from females but had minimal effect on the placentas from males. The full list and information of differentially expressed genes for female placentas in reduced Trp dams vs. control Trp dams is provided in Supplementary File S2, and those for male placentas in reduced Trp vs. control Trp dams is provided in Supplementary File S3. The most up- and downregulated transcripts based on fold-change for female placentas are shown in Table 1, with more details on the one upregulated transcript in male placentas from reduced Trp dams provided in Table 2. These tables also include general functions for these genes.

#### 3.4.3. Target Enrichment and Predicted Diseases/Pathways in Placentas Based on Maternal Trp Dietary Status and Conceptus Sex Differences

**Figure 4:**
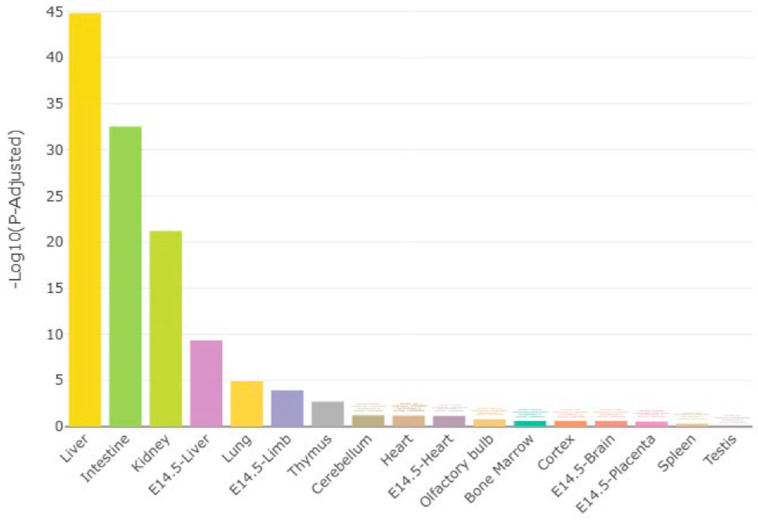
Tissue enrichment or genes that showed differential expression in female placentas derived from conceptuses from reduced Trp dams vs. female placentas derived from conceptuses from control Trp dams. (Tissue enrichment for differentially expressed genes (DEGs) in female placentas for this comparison. For the female placenta, the differentially expressed transcripts were associated with organs regulating nutrient acquisition and metabolism, including the liver, intestine, and kidney.

The TissueEnrich program [52] was used to assess which organs of the mouse have an abundance of transcripts for DEGs in female placentas. Such analysis was not performed for the male placentas as only a few genes were identified to be differentially expressed for the male placental comparison. For female placentas from reduced Trp dams vs. control Trp dams, the differentially expressed transcripts were surprisingly not enriched in the placenta but rather in organs associated with nutrient acquisition and metabolism, including the liver, intestine, and kidney (Figure 4). For male placentas, there were insufficient differentially expressed genes to run this analysis.

**Figure 5:**
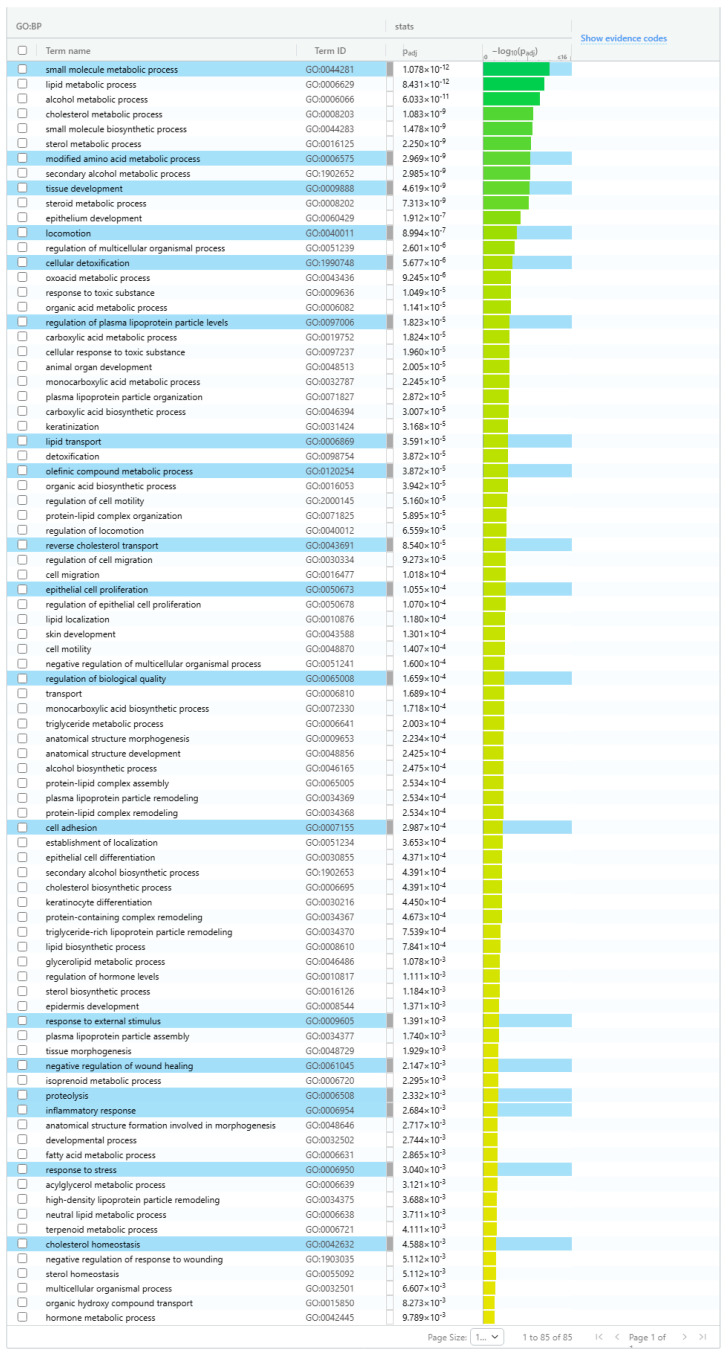
GO BPs associated with differentially expressed transcripts in female placentas derived from reduced Trp dams vs. female placenta from control Trp dams. GO MF pathways associated with differentially expressed transcripts in female placentas include those associated with small molecular metabolic processes, modified amino acid metabolic processes, cell differentiation, regulation plasma lipoprotein particle levels, lipid transport, olenic compound metabolic process reverse cholesterol transport, epithelial cell proliferation, cell adhesion, proteolysis, inflammatory response, response to stress, and cholesterol homeostasis Other pathways are also shown in this figure.

**Figure 6:**
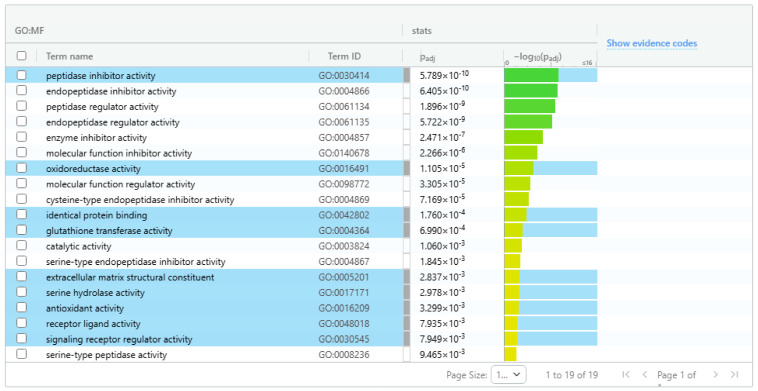
GO MP pathways associated with DEGs in female placentas from reduced Trp dams vs. control Trp dams. GO MF pathways associated with differentially expressed transcripts in female placentas include those associated with peptidase inhibitor activity, oxidoreductase activity, protein binding, glutathione transferase activity, serine hydroslase activity, antioxidant activity, and signaling receptor activity.

GO biological processes (BPs) associated with differentially expressed transcripts in female placentas include those associated with small molecular metabolic processes, modified amino acid metabolic processes, cell differentiation, regulation plasma lipoprotein particle levels, lipid transport, olenic compound metabolic process reverse cholesterol transport, epithelial cell proliferation, cell adhesion, proteolysis, inflammatory response, response to stress, and cholesterol homeostasis (Figure 5). GO molecular function (MF) pathways associated with differentially expressed transcripts in female placentas include those associated with peptidase inhibitor activity, oxidoreductase activity, protein binding, glutathione transferase activity, serine hydroslase activity, antioxidant activity, and signaling receptor activity (Figure 6). GO cellular component (CC) pathways associated with differentially expressed transcripts in female placenta include those associated with the apical part of the cell, intermediate filament, and extracellular region (Figure S4). For the male placental comparison, there were insufficient gene expression differences to determine GO pathways that are likely to be functionally enriched.

#### 3.4.4. Differentially Expressed Genes in Brains Based on Maternal Trp Dietary Status and Sex Differences

The full list and information of DEGs for female fetal brain samples in reduced Trp dams vs. control Trp dams are provided in Supplementary File S4. No differentially expressed genes were identified in male brains from conceptuses derived from reduced Trp dams vs. control Trp dams. Select up- and downregulated transcripts based on fold-change for female brains are shown in Table 3. This table includes general functions for these genes.

#### 3.4.5. Target Enrichment and Predicted Diseases/Pathways in Brains Based on Maternal Trp Dietary Status and Sex Differences

**Figure 7:**
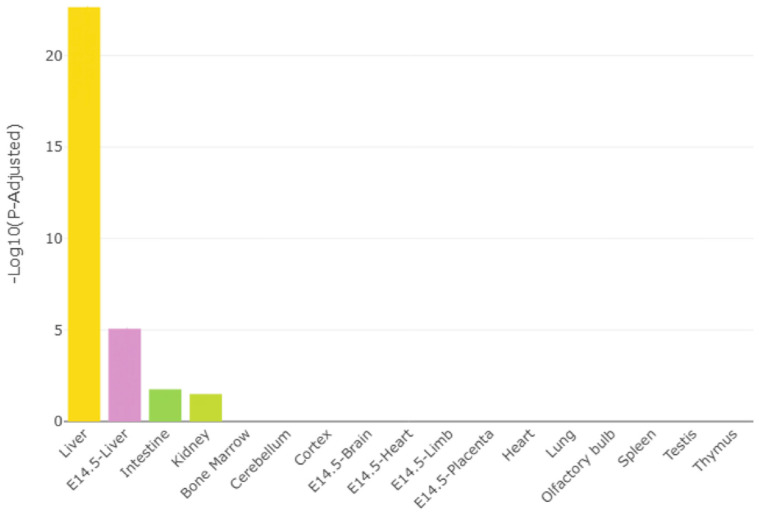
Tissue enrichment or genes that showed differential expression in female placentas derived from conceptuses from reduced Trp dams vs. female placentas derived from conceptuses from control Trp dams. For the female fetal brains from reduced Trp dams vs. female fetal brains from control Trp dams, the differentially expressed transcripts are primarily expressed in the liver, intestine, and kidney.

The TissueEnrich program [52] was used to determine which organs of the mouse have an abundance of transcripts for transcripts differentially expressed in female and male brains. For the female fetal brains from reduced Trp dams vs. female fetal brains from control Trp dams, the differentially expressed transcripts are primarily expressed in the liver, intestine, and kidney (Figure 7).

**Figure 8:**
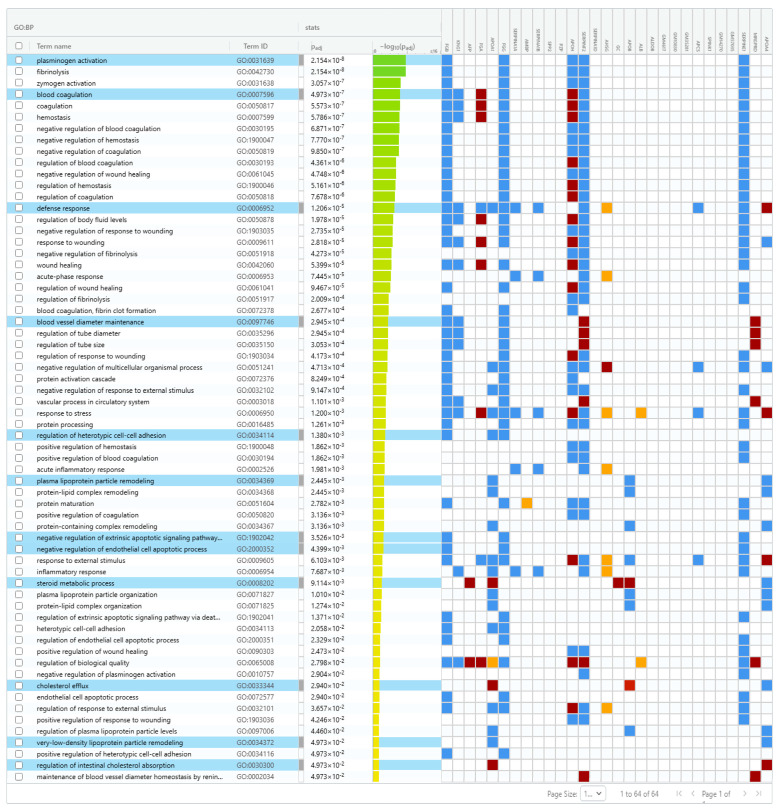
GO BP pathways associated with DEGs in female fetal brains from reduced Trp dams vs. control Trp dams. GO BP processes associated with differentially expressed genes for the female brain comparison include blood vessel diameter maintenance, plasma lipoprotein particle remodeling, plasminogen activation, blood coagulation, defense response, regulation of heterotypic cell–cell adhesion, negative regulation of endothelial cell apoptotic processes, steroid metabolic processes, cholesterol efflux, very reduced density lipoprotein particle remodeling, and regulation of intestinal cholesterol absorption. Other pathways are also shown in this figure.

**Figure 9:**
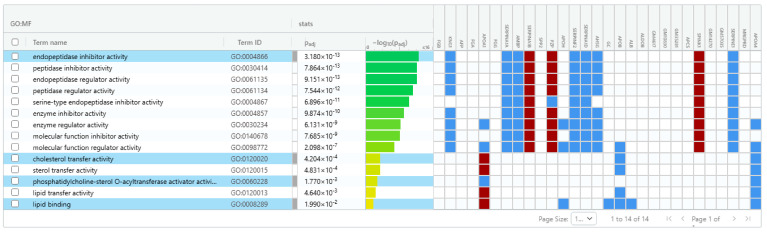
GO MF pathways associated with DEGs in female fetal brains from reduced Trp dams vs. control Trp dams. GO MF processes associated with transcripts that differ between female fetal brains from conceptuses derived from reduced Trp dams vs. those from control dams include endopeptidase inhibitor activity, cholesterol transfer activity, phosphatidylcholine-sterol-O-acyltransferase activity activation, and lipid binding. Other pathways are also shown in this figure.

GO BP processes associated with differentially expressed genes for the female brain comparison include blood vessel diameter maintenance, plasma lipoprotein particle remodeling, plasminogen activation, blood coagulation, defense response, regulation of heterotypic cell–cell adhesion, negative regulation of endothelial cell apoptotic processes, steroid metabolic processes, cholesterol efflux, very reduced density lipoprotein particle remodeling, and regulation of intestinal cholesterol absorption (Figure 8). GO MF processes associated with transcripts that differ between female fetal brains from conceptuses derived from reduced Trp dams vs. those from control dams include endopeptidase inhibitor activity, cholesterol transfer activity, phosphatidylcholine-sterol-O-acyltransferase activity activation, and lipid binding (Figure 9). GO CC processes enriched for differentially expressed transcripts in the female fetal brain comparison include extracellular space and platelet alpha granule (Figure S5). Insufficient transcripts were differentially expressed in male fetal brains to run functional enrichment analysis.

### 3.5. Correlation Analyses of Metabolomics and Transcriptomics Data

**Figure 10:**
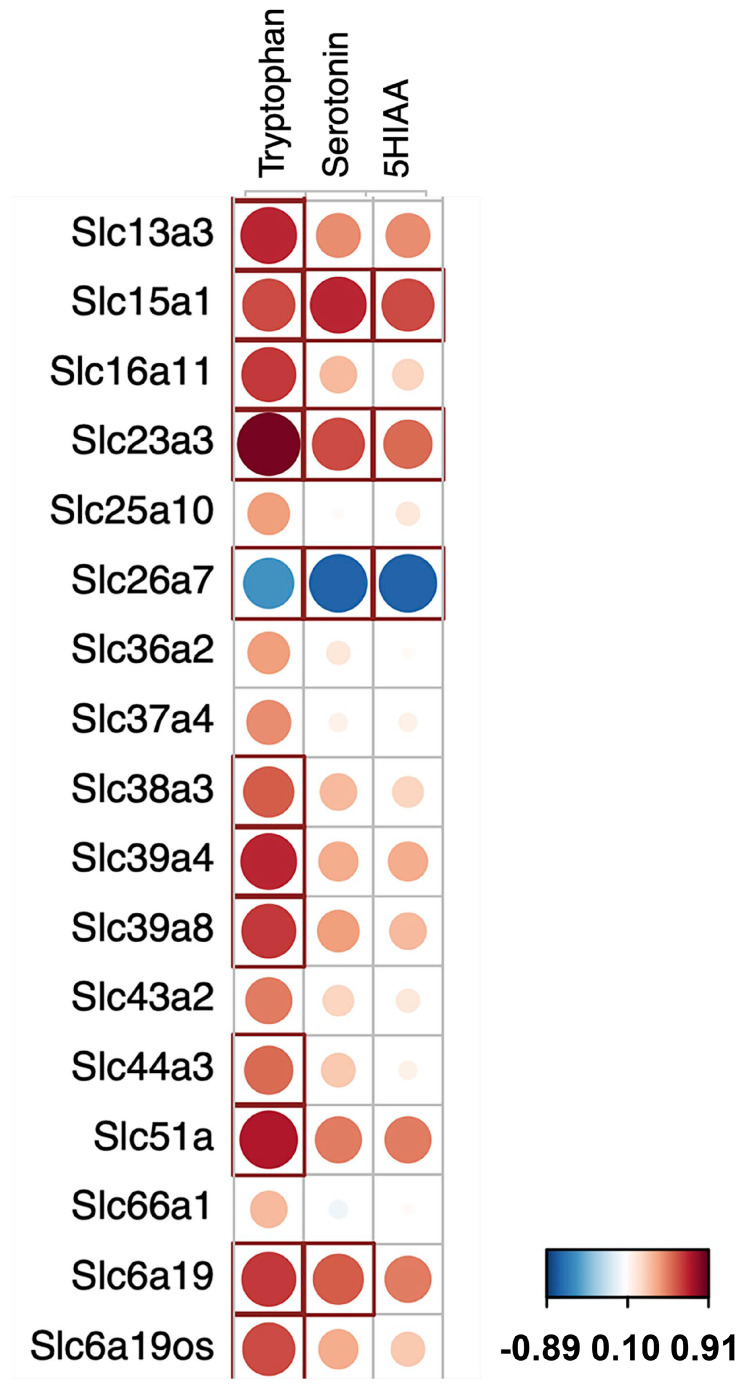
Correlation analysis for select *Slc* genes differentially expressed in female placentas relative to concentrations of Trp, 5-HT, and 5-HIAA in this organ. A complete list of how differentially expressed genes in female placentas correlate with concentrations of these metabolites in this organ is provided in Figure S6. A key to the spectrum of blue to red colors is below the figure. Those correlations that are significant at a *p* value < 0.05 are designated with a red box.

To determine whether associations existed between the amount of Trp, 5-HT, and 5-HIAA within the fetal placenta and brain samples, correlation analyses were performed. In these diagrams, negative correlation analyses are indicated in blue, whereas positive correlations are demarcated in red. A key to the spectrum of blue to red colors is below each of the figures. Those correlations that are significant at a *p* value < 0.05 are designated with a red box. The full list of correlation analyses for differentially expressed genes in female placentas from reduced Trp dams vs. female placentas from control Trp dams is shown in Figure S6. Correlation analyses for differentially expressed genes in male placentas from reduced Trp dams vs. male placentas from control Trp dams is shown in Figure S7. Of the 549 differentially expressed transcripts in the female placental comparison, several of these were positively or negatively associated with amounts of Trp, 5-HT, and 5-HIAA within this organ. Notable associations include *Pomc* and many of the *Serpin* transcripts positively correlated with amounts of Trp in the female placenta (Figure S6). As shown in Table 1, several *Slc* transporter forms showed differential expression in female placentas from reduced Trp dams vs. those from control Trp dams. Figure 10 reveals that many of the *Slc* transporters significantly correlated with Trp and in some cases also with amounts of 5-HT and 5-HIAA in the female placenta. Almost all these linkages were a positive association, with the one exception being *Slc26a7*, which was upregulated in placentas from conceptuses derived from reduced Trp dams, and this transcript was inversely correlated with the amount of Trp, 5-HT, and 5-HIAA in placentas from female conceptuses. This transporter shuttles chloride, bicarbonate, sulfate, and iodide across cell membranes. *Slc6a19* (a Trp transporter that showed reduced expression in female placentas from reduced Trp dams) was positively associated with the amount of Trp and 5-HT in this organ. *Slc6a4,* which encodes SERT, was downregulated in the placentas from these females, and the expression of this gene was positively associated with amounts of Trp, 5-HT, and 5-HIAA within the placenta. For male placentas, *Car6* showed increased expression in conceptuses derived from reduced Trp dams, and this transcript was negatively associated with amounts of 5-HT and 5-HIAA in this organ (Figure S7).

**Figure 11:**
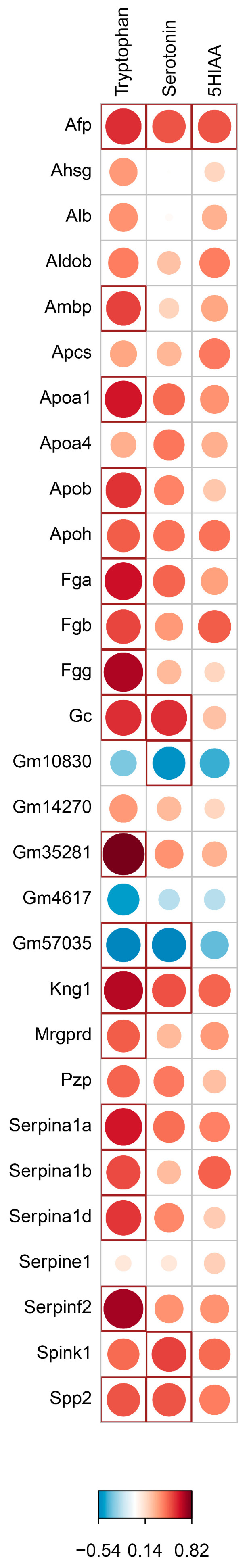
Correlation analysis for differentially expression genes in female fetal brains from low Trp vs. control dams and relation to concentrations of Trp, 5-HT, and 5-HIAA in this organ. A key to the spectrum of blue to red colors is below the figure. Those correlations that are significant at a *p* value < 0.05 are designated with a red box.

For the female fetal brain, expression of *Afp*, *Ambp*, *Apoa1*, *Apob*, *Apoh*, *Fga*, *Fgb*, *Fgg*, *Gc*, *Kng1*, *Serpina1a*, *Serpina1b*, *Serpina1d*, *Serpinf2*, and *Spp2* were positively correlated with amounts of Trp in this organ (Figure 11). *Afp* expression was also positively correlated with amounts of 5-HT and 5-HIAA in the female fetal brain. Additionally, the amount of 5-HT in this organ was positively linked with expression for *Gc*, *Kng1*, *Spink1*, and *Spp2*. No genes were differentially expressed in male fetal brains to run this correlation analysis.

## 4. Discussion

In the current work, we assessed how reduction in Trp in the diet fed to pregnant mice impacts the placenta–brain axis. As part of these studies, concentrations of Trp, 5-HT, and 5-HIAA were examined in the maternal serum and uterine tissues. Secondly, the concentrations of these metabolites were measured in the placentas and fetal brains of female and male conceptuses. Additionally, current studies explored how this reduction in dietary maternal Trp influences transcriptomic profiles in the female and male placenta and fetal brain. To our knowledge, these are the first studies to perform a comprehensive examination of how reduction in one maternal amino acid can affect her developing offspring. Trp is an essential precursor to 5-HT that is required for normal placental and fetal brain development. At the outset, we predicted that dams on the reduced Trp diet would exhibit reductions in circulating and uterine concentrations of Trp, 5-HT, and 5-HIAA. While there was a trend towards reduction in Trp in dams on the reduced Trp diet compared to those on the control Trp diet, it was not statistically significant (6.96 ± 2.83 ng/mL vs. 13.05 ± 2.45 ng/mL, respectively, *p* = 0.07). These studies tested 4–6 replicates/groups, which, based on our power analyses and previous work, should have been sufficient to yield significant differences if they were to be observed. It is possible that had we tested more females per group, statistically significant differences between these two groups would have been detected. The same is true for 5-HT, where there was a trend towards a reduction in the reduced Trp dams vs. the control Trp dams of 0.803 ± 0.59 vs. 1.62 ± 0.75, respectively, *p* = 0.23. No overt differences in circulating concentrations for 5-HIAA, a metabolite of 5-HT, were detected between these two groups, which could reflect reduced metabolism of 5-HT in the dams on the reduced Trp diet. No obvious differences were detected for these three metabolites in uterine tissue. The amounts of Trp and 5-HT in uterine tissue for both groups of dams were significantly less than those detected in the placenta and fetal brain, suggesting that these metabolites do not collect to a great extent in this maternal tissue and/or readily transfer across to the placenta where they are needed.

While reductions in maternal Trp did not significantly alter Trp, 5-HT, and 5-HIAA concentrations in the maternal serum and uterine tissue, such reductions led to dramatic reductions in these three metabolites in the female and male placenta and fetal brain tissue. Intraamniotic injection of LPS into pregnant rats has previously been shown to reduce tryptophan hydroxylase (TPH) expression by the placenta and lead to decreased placental and fetal brain concentrations of 5-HT [15]. Conversely, earlier work with pregnant rats showed that excess maternal Trp supplementation increases fetal neural Trp and 5-HT concentrations [57]. This study also reported that Trp concentrations in fetal brains are considerably higher than those in the fetus and placenta [57]. In the current work, we also found that Trp concentrations in female and male fetal brain tissues from control dams were greater than those in the female and male placentas from control Trp dams. However, 5-HT concentrations were about 10-fold higher in female and male placentas from control dams compared to female and male fetal brain tissue from these same dams, which assumingly relates to the early stage of brain development by this point. Later in gestation, it is likely that similar or even greater concentrations of 5-HT would be evident in the brain relative to the placenta, especially given the accrual of Trp that can be metabolized to 5-HT in various brain regions.

The above findings also raise the question as to why no differences in maternal Trp and 5-HT serum and uterine concentrations were evident between the reduced Trp dams compared to control Trp dams. One possibility is that reduced amounts of Trp and possibly 5-HT are shuttled from maternal blood to the placenta. In support of this notion, the female placenta from reduced Trp dams had reduced expression for the transporters for Trp and 5-HT (*Slc6a19* and *Slc6a4*, respectively). Yet, no change in these transporters was evident in male placentas from these dams, but they also exhibited reductions in both metabolites in the placenta and fetal brain. It may be that other transporters for these metabolites were affected. It could also be hypothesized that the mother can regulate delivery of such nutrients to the conceptus, and in times of deficits, reduces the transfer of nutrients in short supply. The underpinning mechanisms favoring such maternal homeostasis over her conceptuses’ health can only be speculated upon. Conceivably, the dam might reduce blood flow to the placenta, which would effectively reduce delivery of several nutrients. More targeted approaches that could be envisioned are the ability of the dam to suppress or epigenetically regulate placenta transporters for Trp and 5-HT. The latter possibility invokes a dynamic interplay between the mother and placenta and might apply broadly to placental transfer of other nutrients. Providing isotopically labeled Trp and 5-HT to the dam might be useful to elucidate the rate of transfer of these metabolites across the placenta and distribution to the fetal brain and other organs.

Female mice that lack a Trp diet can conceive but then proceed to abort their fetuses due to implantation failure [7]. It is uncertain whether this compromised fecundity is maternal in origin or due to improper conceptus signaling. In the current work, female mice on the low Trp diet were able to become pregnant and had a similar number of conceptuses as dams on the control Trp diet, but the reduced amounts of Trp and 5-HT in the placenta and fetal brain likely compromised development of these organs.

Conflict exists as to whether the placenta internalizes maternal 5-HT or synthesizes itself from maternal 5-HT. Whether the current studies have provided any insight into this matter remains uncertain. It is clear that the placenta has higher concentrations of 5-HT than uterine tissue, but the placenta could act as a sink to collect and eventually distribute to the fetus, in particular the brain, 5-HT derived from maternal sources and/or that produced locally. Reductions in maternal Trp might lead to decreased production of maternal 5-HT and/or placental-derived 5-HT. Potential experiments that could be conceived to settle this controversy would be to provide pregnant mice labeled Trp in reduced, controlled amounts, and excess concentrations and then determine the concentrations of labeled Trp in maternal serum, uterine tissue, placenta, and fetal brain. Alongside these studies, Trp with a different radioactive label could be injected into amniotic cavity. Assumingly, if trophoblasts produce 5-HT, more of the neurotransmitter would carry the label derived from intraamniotic injection of Trp, whereas if the placenta accrues maternal 5-HT, greater amounts of 5-HT would be labeled with the Trp form provided to the dam.

Regardless of the source, Trp and 5-HT are essential for normal placental and fetal brain development and function. Transgenic mice lacking *Tph1* and *Slc6a4/SERT* reveal the important roles 5-HT exerts directly in the placenta. Whole-body *Slc6a4*/SERT KO mice show reduced placental weight at embryonic age (E) 18 compared to controls with marked apoptosis and areas of necrosis, hemorrhage, and fibrosis in the one of the placental layers (spongiotrophoblast) [22]. Placentas from whole-body *Tph1* KO mice also exhibited an increase in apoptosis and decreased proliferation but to a lesser extent than the whole-body *Slc6a4*/SERT KO mice [22]. In our own studies, placentas at E12.5 from *Slc6a4*/SERT KO mice had changes in trophoblast cell populations relative to WT [58]. In these studies, we used RNAseq analysis to determine how whole-body deletion of this gene affected transcriptome profiles in the placenta and discovered that relative to WT counterparts, *Slc6a4*/SERT KO had increased expression of genes associated with intestinal functions, including nutrient sensing, uptake, catabolism, and blood clotting [58]. Pups of whole-body *Slc6a4*/SERT KO show normal early development but as they age, they begin to display autism spectrum disorder (ASD)-like behavioral deficits [59,60,61].

To understand further the importance of placental *Slc6a4* on the placenta–brain axis, we created a mouse that lacked this gene in select trophoblast cells of the placenta (parietal trophoblast giant cells, pTGC) [62]. Selective deletion of *Slc6a4* in pTGC of the mouse placenta resulted in sex-dependent changes in the placenta, including reduced number of pTGC and transcriptomic alterations in the placenta that were most overwhelming in the placenta of female offspring [62]. Further, ablation of this gene in the placenta impacted fetal brain development, including altering epinephrine and metanephrine in the fetal brain and resulting sexually dimorphic gene expression changes in this organ [62].

The second part of the current studies was to determine how reductions in maternal Trp mediate transcriptomic changes in the placenta and fetal brain of female and male conceptuses. Sex differences in risk were predicted to occur. However, we did not anticipate how skewed such differences in vulnerability would be in response to lowering a single amino acid in the maternal diet. Importantly, these studies are the first to provide robust evidence that the female placenta and fetal brain demonstrate striking differences in gene expression changes in response to reduce maternal Trp, but such changes are negligible in male placentas and fetal brains. Both in the female placenta and brain for conceptuses derived from Trp dams, gene expression changes align to nutrient acquisition and metabolism pathways. In the case of the female placenta from this group, several nutrient and metabolite transporters showed reduced expression, including *Slc6a19* that transfers Trp and other neutral amino acids and *Slc6a4* that binds and transfers 5-HT from maternal blood to the placenta. Correlation analyses revealed a positive association between amounts of Trp and 5-HT in the placenta and expression of these and other *Slc* transporters. The potential significance and directionality of this linkage remain to be determined.

We, and others, have previously reported that the placentas of female conceptuses seem to be exquisitely sensitive to changes in maternal diet and genetic status and other in utero environmental changes [36,63,64,65,66,67,68,69]. Potential mechanisms contributing to sex-dependent differences in placental gene expression have been discussed previously [70]. At ~12.5 dpc, there is minimal differentiation of the genital ridge into an ovary or testis. Therefore, gonadal steroid hormones, namely estrogen and testosterone, likely exert minimal contribution to sexually dimorphic differences in the placenta and fetal brain observed in the current work. Dogma suggests that the paternal X chromosome is selectively inactivated in female placentas, but some genes on the X chromosome might escape X inactivation. The X chromosome encodes several genes associated with nutrient acquisition and metabolism.

Epigenetic changes, including in DNA methylation patterns, histone protein modifications, and expression of miRNAs, might also account for sex-dependent differences in the placenta and fetal brain following changes in maternal diet [35,39,70,71,72,73,74,75]. Sex-dependent epigenetic changes in the placenta have been postulated to underly maternal nutritional programming of offspring health and disease [76]. As such, future work in this area would be aided by a comprehensive dataset for sex-dependent datasets for DNA methylome, histone protein modifications, and miRNA profiles in the placenta and fetal brain at various stages and under varying maternal nutritional conditions. Commonalities with altered maternal nutritional status should be explored. It would also be helpful to determine the degree to which sex-dependent differences in fetal brain gene expression patterns persist in different brain regions as the offspring mature and how such gene expression changes correlate with behavioral parameters.

In future studies, we seek to determine the basis for such sex differences, including potential mechanisms listed above. The long-term implications of such early sex differences to maternal dietary changes merit further pursuit. While the placenta is an ephemeral organ, how it responds to in utero environmental challenges can affect development of the fetal brain and other organ systems. Correspondingly, neurobehavioral disorders and other diseases might trace their ontogeny back to placental responses to in utero environmental alterations. Whether the transcriptomic changes in the placenta and fetal brain of female conceptuses in response to reductions in maternal Trp convey protection against this seemingly unfavorable in utero change or are maladaptive remains to be determined.

Studies with Sprague-Dawley rats revealed that male offspring from dams providing overnutrition had an increase in metabolites involved in Trp and linoleic acid metabolism, and these elevations correlated with an increased risk of metabolic and neurodegenerative diseases [77]. Thus, sex-dependent differences in the placenta and fetal brain for other metabolites should also be investigated in follow-up work.

Potential limitations of the study are that only one timepoint in gestation was examined. It is possible that more pronounced changes in male placentas and fetal brains would become evident later in gestation. Gene expression changes in the female brain might also become more pronounced in late gestation. It would also be of interest to confirm that the mice on the reduced Trp diet have normal parturition and lactation. Future studies will also examine protein expression patterns for genes, such as SLC6A4 (SERT) and SLC6A19 that showed decreased transcript expression in female placentas from dams on the reduced Trp diet. Further, we will investigate how this maternal dietary reduction impacts placental and brain histomorphology in female and male conceptuses. Lifelong health effects, especially neurobehavioral traits, should be assessed for male and female offspring born to pregnant dams provisioned with a reduced Trp diet. Potential disruptions in neurobehavioral traits might trace their origins back to deficiencies in Trp and 5-HT concentrations in the placenta and fetal brain, as well as transcriptomic changes in these organs, especially in females.

## 5. Conclusions

Current studies reveal that dams fed a low Trp diet exhibit similar serum and uterine Trp and 5-HT concentrations as those provided a control Trp diet. However, male and female conceptuses derived from dams on the reduced Trp diet have reduced amounts of Trp and 5-HT in the placenta and fetal brain compared to conceptuses derived from dams on the control diet. Reductions in dietary maternal Trp result in striking sexually dimorphic gene expression changes, especially in the female placenta. Gene expression changes in the female placenta are associated with amounts of Trp and 5-HT in this organ. Transporters for Trp and 5-HT (*Slc6a19* and *Slc6a4*, respectively) show reduced expression in female placentas for conceptuses derived from low Trp dams, and expression of these transporters and other *Slc* transporters are positively correlated with Trp and 5-HT within the placenta. Findings assumingly have clinical relevance to pregnant women as they show even subtle reductions in one amino acid can alter nutrient delivery to the placenta and fetal brain and thereby might profoundly influence offspring development and possible risk for later neurobehavioral disorders. Results also suggest the placenta and fetal brain of female conceptuses might be vulnerable to in utero nutrient changes. The current work suggests cause for concern that even slight changes in maternal diet can cause sex-dependent differences in the placenta and fetal brain, but the clinical implications for such changes remain to be elucidated.

## Figures and Tables

**Table 1:** Select up- and downregulated transcripts in female placentas from reduced Trp dams vs. control Trp dams with a *p*-adj (q- value) ≤ 0.05.

Gene Symbol	Fold Change (GF vs. MPF Dams)	*p*-adj (q Value)	General Function of the Encoded Protein
***Select Downregulated Transcripts***			
*Wnt10a*	0.127	0.000298	Involved in the Wnt signaling pathway and promotes placenta growth, differentiation, and invasion of trophoblast cells.
*Slc13a3*	0.129	0.048659	Encodes for Sodium/Dicarboxylate Cotransporter 3 (NaDC3) and, in trophoblast cells, mediates the uptake and transfer of metabolic intermediates, such as succinate and citrate, between the mother and fetus.
*Apoe*	0.141	8.17 × 10^−5^	Encodes for apolipoprotein E, which is crucial for lipid/cholesterol transport in the placenta.
*Slc23a3*	0.155	0.023438	Encodes for a hypoxanthine transporter, which is essential for cellular energy pathways and nucleic acid synthesis.
*Slc6a19*	0.306	0.006882	Encodes for System B(0) neutral amino acid transporter 1 (B^0^AT1), which regulates absorption of primarily tryptophan and other neutral amino acids.
*Slc36a2*	0.322	0.023087	Encodes for an electrogenic proton/amino acid symporter that mediates the transfer of other small neutral amino acids, such as glycine, alanine, and proline, from the mother to the fetus.
*Slc6a4*	0.421	0.041137	Encodes for the serotonin transporter (SERT), which regulates internalization of maternal 5-HT by the placenta. It is essential for normal placental growth, nutrient acquisition, and fetal brain development.
***Select Upregulated Transcripts***			
*Nol4*	14.418	0.00023	Encodes for a protein that functions as an RNA-binding protein within the cell nucleolus.
*Car6*	9.266	3.48 × 10^−5^	Encodes for carbonic anhydrase VI that catalyzes the hydration of carbon dioxide.
*Cyp1a1*	7.422	0.002075	Encodes for Cytochrome P450 1A1 that metabolizes environmental toxins, drugs, and dietary chemicals. In humans, elevated expression of this enzyme is linked with intra-uterine growth restriction, lower birth weight, and premature delivery.
*Slc26a7*	2.976	0.042201	Encodes for a transmembrane transporter for chloride, sulfate, and iodide. In the placenta, it is essential for sulfate transport from mother to fetus.
*Hsd17b7*	2.788	0.000842	Encodes for an enzyme that converts estrone into estradiol, and it is upregulated in trophoblast cells.
*Cyp2ab1*	2.716	0.000481	Encodes for a cytochrome P450 monooxygenase that is abundant in placental tissue, where it regulates xenobiotic and steroid metabolism.

**Table 2:** Known upregulated transcript in male placentas from reduced Trp dams vs. control Trp dams with a *p*-adj (q- value) ≤ 0.05.

Gene Symbol	Fold Change (GF vs. MPF Dams)	*p*-adj (q Value)	General Function of the Encoded Protein
***Select Upregulated Transcripts***			
*Car6*	10.801	7.34 × 10^−12^	Encodes for carbonic anhydrase VI that catalyzes the hydration of carbon dioxide.

**Table 3:** Select up- and downregulated transcripts in female fetal brain samples from reduced Trp dams vs. control Trp dams with a *p*-adj (q- value) ≤ 0.05.

Gene Symbol	Fold Change (GF vs. MPF Dams)	*p*-adj (q Value)	General Function of the Encoded Protein
***Select Downregulated Transcripts***			
*Ambp*	0.054	1 × 10^−5^	Encodes for alpha-1-microglobulin/bikunin precursor, which modulates antioxidant pathways and peripheral immune responses within the brain.
*Apoh*	0.08	2.92 × 10^−5^	Encodes for apolipoprotein H that binds to lipoproteins and phospholipids; both of these biomolecules regulate lipid transport, blood clotting, and immune responses within the brain and other organs.
*Afp*	0.099	3.31 × 10^−10^	Encodes for alpha-fetoprotein (AFP) that protects the developing fetal brain against excess maternal estrogens.
*Apob*	0.102	0.003115	Encodes for apolipoprotein B, which binds to the low-density lipoprotein (LDL) form of cholesterol and can influence brain health.
*Apoa4*	0.14	0.04041	Encodes for apolipoprotein A-IV and expression in the hypothalamus and brainstem and regulates appetite and overall brain health.
*Apoa1*	0.173	3.48 × 10^−9^	Encodes for apolipoprotein A-I that binds to high-density lipoprotein (HDL) cholesterol, which can exert neuroprotective roles, including immunosuppression.

## Data Availability

RNA sequencing data have been deposited and are publicly available in the Gene Expression Omnibus under accession: GSE330442 (accessed on 2 August 2026).

## Supplementary Materials

The following supporting information can be downloaded at: https://www.mdpi.com/article/10.3390/nu18152575/s1. Figure S1. Comparison of pre and post-body weight and litter size in reduced and control Trp pregnant female mice. (A) Body weight. (B) No differences in litter size were noted between pregnant female mice on the reduced Trp diet compared to those on the control Trp diet (8.17 ± 0.64 vs. 8 ± 1.13 conceptuses, respectively, *p* = 0.90). Figure S2. 2D PCA plot of RNAseq results from placenta and fetal brain samples from reduced Trp (0.1%) and control Trp (0.2%) diet. (A) 2D PCA plot of RNAseq results for female placenta from conceptuses derived from reduced Trp (0.1%) vs. female placenta from conceptuses derived from control Trp (0.2%) dams. (B) 2D PCA plot of RNAseq results for male placenta from conceptuses derived from reduced Trp (0.1%) vs. male placenta derived from conceptuses control Trp (0.2%) dams. (C) 2D PCA plot of RNAseq results for female fetal brain from conceptuses derived from reduced Trp (0.1%) vs. female fetal brain from conceptuses derived from control Trp (0.2%) dams. (D) 2D PCA plot of RNAseq results for male fetal brain from conceptuses derived from reduced Trp (0.1%) vs. male fetal brain from conceptuses derived from control Trp (0.2%) dams. PERMANOVA values for female and male placental comparisons were 0.05 and 0.23, respectively. PERMANOVA values for female and male fetal brain comparisons were 0.04 and 0.26, respectively. Thus, there was significant separation for the female group com-parisons but not for the male group comparisons. Figure S3. Venn diagram for overlap in transcripts when comparing differentially expressed genes in female placenta derived from reduced Trp vs. control Trp dams compared to differentially expressed genes in male placenta derived from reduced Trp vs. control Trp dams. Of the 549 known differentially expressed transcripts in female placenta compared to the two known differentially expressed genes in male placenta, both genes differentially expressed in the male (Car6 and 1810073O08Rik) overlapped when comparing reduced Trp vs. Control Trp placental samples. List of genes that are solely differentially expressed in female placenta from reduced Trp vs. Control Trp dams in column A, and list of differentially expressed genes that overlap in female and male comparisons in column B. Figure S4. GO CC pathways associated with DEG in female placenta from reduced Trp dams vs. control Trp dams. GO CC pathways associated with differentially expressed transcripts in female placenta include those associated with apical part of the cell, intermediate filament, and extracellular region. Other cellular components/pathways are shown in this figure. Figure S5. GO CC Pathways associated with DEG in female fetal brain from reduced Trp dams vs. control Trp dams. GO CC processes enriched for differentially expressed transcripts in female fetal brain comparison include extracellular space and platelet alpha granule. Other pathways are also shown in this figure. Figure S6. Correlation analyses of differentially expressed genes in female placenta from reduced Trp dams vs. control Trp dams and concentrations of Trp, 5-HT, and 5-HIAA in this organ. This diagram included the full list of DEGs in female placenta and their association with these metabolites. A key to the spectrum of blue to red colors is below the figure. Those correlations that are significant at a *p* value < 0.05 are designated with a red box. Figure S7. Correlation analyses of differentially expressed genes in male placenta from reduced Trp dams vs. control Trp dams and concentrations of Trp, 5-HT, and 5-HIAA in this organ. This diagram included the full list of DEGs in female placenta and their association with these metabolites. A key to the spectrum of blue to red colors is below the figure. Those correlations that are significant at a *p* value < 0.05 are designated with a red box. Table S1. Dietary composition of reduced Trp (0.1%) diet that was provided prior to conception and during pregnancy to the mice in this study. Table S2. Dietary composition of control Trp (0.2%) diet that was provided prior to conception and during pregnancy to the mice in this study. Table S3. Number of raw reads, mapped reads, and % mapped reads from RNAseq results from placenta and fetal brain samples from pregnant female mice on the control Trp diet (0.2%) and reduced Trp (0.1%) diet. Table S4. Tryptophan (Trp), serotonin (5-HT), and 5-HIAA concentrations in maternal serum and uterine tissue. Concentrations are on ng/μL for maternal serum and ng/mg for uterine tissue. Table S5. Tryptophan (Trp), serotonin (5-HT), and 5-HIAA concentrations in placenta and fetal brain samples from female. Concentrations are in ng/mg tissue. Supplementary File S1. Venn comparison results for overlap in transcripts when comparing differentially expressed genes in female placenta derived from reduced Trp vs. control Trp dams compared to differentially expressed genes in male placenta derived from reduced Trp vs. control Trp dams. Of the 549 known differentially expressed transcripts in female placenta compared to the two known differentially expressed genes in male placenta, both genes differentially expressed in the male (Car6 and 1810073O08Rik) overlapped when comparing reduced Trp vs. Control Trp placental samples. List of genes that are solely differentially expressed in female placenta from reduced Trp vs. Control Trp dams in column A, and list of differentially expressed genes that overlap in female and male comparisons in column B. Supplementary File S2. Differentially expressed genes in female placenta from reduced Trp dams vs. female placenta from control Trp dams. Supplementary File S3. Differentially expressed genes in male placenta from reduced Trp dams vs. male placenta from control Trp dams. Supplementary File S4. Differentially expressed genes in female fetal brain from reduced Trp dams vs. female fetal brain from control Trp dams.
