# Supplementary material for: Diminished Maternal Tryptophan Leads to Sexually Dimorphic Differences in the Placenta–Brain Axis
Source: Nutrients. 2026 Aug 6;18(15):2575. doi: 10.3390/nu18152575 (PMC13468233; doi:10.3390/nu18152575)
Supplement: Supplementary file 1 [file nutrients-18-02575-s001.zip › Supplementary Figure S6_7_26_26.pdf]

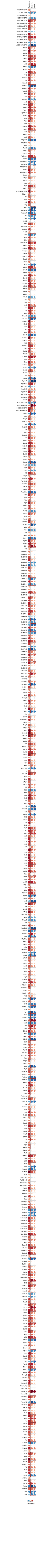
Tryptophan
Serotonin
5HIAA
0610005C13Rik
1110002E22Rik
1810044D09Rik
1810073O08Rik
2810459M11Rik
3300005D01Rik
4930438A08Rik
4930511P09Rik
4932415M13Rik
4933427E11Rik
5730419F03Rik
5930403N24Rik
6720407P12Rik
A230001M10Rik
Abcc8
Ablim3
Acat2
Acox2
Acsm1
Acta1
Add2
Adgrl1
Adrb2
Afp
Ahsg
Akr1c13
Alb
Aldh1a1
Aldh1a3
Aldh1l1
Alox12b
Amn
Ang
Ankrd45
Ano9
Anxa6
Apoa1
Apoa4
Apobec2
Apoc1
Apoe
Apof
Apoh
Apol7a
Apom
Apon
Aqp11
Aqp8
Arhgap39
Arhgap40
Artn
Asb16
Asgr2
Asprv1
Atp6v0a4
Atp8b2
AU015791
B4galnt2
BB123696
Bbox1
BC028471
Bcam
Bglap3
Bicdl2
Bnipl
Bst1
Btnl12
C130074G19Rik
C1qtnf4
C2
C4b
C8b
C8g
Cabp7
Cacna1b
Cacna1h
Car2
Car6
Cavin3
Cblc
Ccdc198
Ccdc80
Ccl17
Cd69
Cdh16
Cdsn
Ceacam15
Cela1
Cela2a
Cenpm
Cercam
Cfap221
Cgref1
Chi3l1
Chrdl2
Chst5
Chsy3
Cideb
Cited4
Ckmt1
Clca1
Clca4b
Cldn2
Cldn3
Cldn6
Cldn9
Clhc1
Clic3
Cln6
Clrn3
Clu
Cnfn
Cnn1
Col11a1
Col15a1
Col6a1
Col6a2
Cpa4
Cpb2
Cpn1
Cpne5
Cpxm2
Creb3l3
Crip1
Crybb2
Cryl1
Csdc2
Csta1
Cstdc5
Ctsh
Cutal
Cwh43
Cxcl1
Cxcl16
Cxcl17
Cyba
Cyfip2
Cyp1a1
Cyp21a1
Cyp2ab1
Cyp2b19
Cyp2c70
Cyp2d26
Cyp3a13
Cysrt1
D130043K22Rik
D130052B06Rik
D630024D03Rik
D930019O06Rik
Dclk2
Dcxr
Depp1
Dhrs13
Dlg2
Dnajc22
Dpt
Dsg3
Dusp26
Eln
Elovl7
Emp2
Entpd2
Entpd3
Epcip
Ephb6
Ephx1
Ephx2
Ermap
Espl1
Espn
Eya1
F2
Fabp5
Fam20c
Fam210b
Fam25a
Fam3b
Fam83e
Fancd2os
Fcgrt
Fcna
Fdps
Fgf1
Fgfbp1
Flacc1
Fndc9
Foxa3
Fut2
Fxyd1
Gal3st1
Gal3st3
Galm
Gatm
Gcat
Gchfr
Gcnt4
Ggact
Gipc2
Gjb4
Gli2
Gm10051
Gm10288
Gm10638
Gm10736
Gm10848
Gm11100
Gm11827
Gm12295
Gm12349
Gm13216
Gm13431
Gm13605
Gm15344
Gm15536
Gm15867
Gm16410
Gm19619
Gm26717
Gm28884
Gm30794
Gm31126
Gm31718
Gm32727
Gm33694
Gm35268
Gm36972
Gm38273
Gm39770
Gm4065
Gm43375
Gm44617
Gm44850
Gm44867
Gm44873
Gm4651
Gm46603
Gm47815
Gm48635
Gm49282
Gm49643
Gm50288
Gm5478
Gm5617
Gm56508
Gm56938
Gm57165
Gm57435
Gm5781
Gm6093
Gm8801
Gm8834
Gm9347
Gmpr
Gpc3
Gpihbp1
Gprin1
Gpt2
Gpx1
Grb7
Grik3
Gss
Gstm1
Gstm2−ps1
Gstm5
Gstp2
Gstp3
Gstt1
H2−Q10
H2−T15
Hdhd3
Hgfac
Hkdc1
Hmgcs2
Hnf1a
Hnf1b
Hoga1
Hoxb7
Hpn
Hpx
Hrnr
Hsd17b7
Hyi
Ifi27l2b
Ifi30
Ifitm1
Igfbp4
Igsf23
Ihh
Il1r1
Il4ra
Inhca
Ins2
Islr
Itih1
Itk
Kbtbd11
Khk
Kif26a
Klf15
Klk11
Klra4
Klrg2
Kng1
Kng2
Krt1
Krt10
Krt13
Krt14
Krt15
Krt16
Krt17
Krt20
Krt5
Krt6a
Krt6b
Krt77
Krt78
Krtdap
Lamb3
Lcat
Lce1m
Lgals2
Lgals7
Lhfpl7
Lhpp
Lipe
Loricrin
Lpar5
Lrrc66
Lrrn2
Ly6d
Ly6k
Lypd3
M1ap
Mab21l4
Mafa
Magel2
Mal
Man2b2
Map3k21
Map7d2
Mboat1
Mcc
Mfsd13a
Mgst2
Mgst3
Misp
Mkrn2os
Mmp1b
Mpzl2
Ms4a10
Mst1
Mtarc1
Muc13
Muc16
Mvd
Myh14
Myl9
Myo15b
Myo7b
n−R5s194
Naaladl1
Naprt
Nectin4
Ngp
Nkain1
Nkx2−3
Nlrc5
Nmnat2
Nnat
Nol4
Nos1
Nt5dc3
Ntsr1
Nuak1
Nyx
Olah
Olfml1
Olr1
Opn3
Or2t43
Osr1
Pcbd1
Pcsk5
Pcsk9
Pcyox1
Pdzk1ip1
Pecr
Phgr1
Phlda2
Pkib
Pkp1
Pla2g12b
Pla2g2f
Pla2g4d
Pla2g4e
Plgrkt
Pllp
Podxl2
Pomc
Ppbp
Ppp1r14a
Ppp1r14d
Ppp2r2c
Ppt2
Prdm14
Proc
Prph
Prr15l
Prss27
Psapl1
Psat1
Psca
Ptprh
Pwwp2b
Pxmp2
Pycard
Pyy
Pzp
Qprt
Rab25
Rab4a
Rac3
Rarres1
Rbp4
Rdh7
Rec8
Reln
Rimbp3
Rnf186
Rorc
Rpl28−ps1
Rpl36−ps2
Rps13−ps2
Rps18−ps1
Rsph9
S100a3
S100a4
Saa4
Sdsl
Selenbp1
Selenbp2
Sema4a
Sema4c
Sema4d
Sema6d
Serpina10
Serpina1a
Serpina1c
Serpina1d
Serpinb11
Serpinb2
Serpinb5
Serpinf1
Shcbp1l
Sigirr
Slc13a3
Slc15a1
Slc16a11
Slc23a3
Slc25a10
Slc26a7
Slc36a2
Slc37a4
Slc38a3
Slc39a8
Slc51a
Slc6a19
Slc6a19os
Slc6a4
Slfn2
Smim22
Smim24
Smpd3
Snora41
Snord34
Snx20
Soat2
Sod3
Sox11
Spink5
Spint1
Spns2
Spns3
Sprr1a
Sprr1b
Spsb4
Sptlc3
Stfa1
Stfa2
Stfa3
Stox2
Susd2
Sycn
Syne4
Syt13
Sytl3
Tcea3
Tceal5
Tet1
Tgm3
Thbs4
Tiam2
Timd2
Tm4sf5
Tm7sf2
Tmed6
Tmem117
Tmem176a
Tmem176b
Tmem178
Tmem205
Tmem252
Tmem51
Tmem72
Tmprss13
Tmt1b
Tnnc1
Tnxb
Tppp3
Trex2
Trf
Trim15
Trim29
Trim47
Trim63
Trnp1
Tspan13
Ttr
Tvp23a
Uap1l1
Ugt3a1
Upk1a
Upk1b
Upk2
Upk3b
Upk3bl
Vaultrc5
Vil1
Vnn3
Vtn
Wdr90
Wnt10a
Wnt4
Wnt7a
Wnt8a
Zfp385b
Zic5
Zp2
−0.89
0.01
0.91
