# Supplementary material for: Diminished Maternal Tryptophan Leads to Sexually Dimorphic Differences in the Placenta–Brain Axis
Source: Nutrients. 2026 Aug 6;18(15):2575. doi: 10.3390/nu18152575 (PMC13468233; doi:10.3390/nu18152575)
Supplement: Supplementary file 1 [file nutrients-18-02575-s001.zip › Supplementary Material7_25_26.pdf]

Supplementary Material

Diminished Maternal Tryptophan Leads to Sexually Dimorphic Differences in the Placenta-Brain Axis

Rosalind T.B. Herrington[1,2], Zhen Lyu[3,4], Sarah E. Seda[1,5], Emmett E. Boling[1,2], Andrea K. Goldstein[6], Nathan J. Bivens[7], Zhentian Lei[4,8,9], Tanhaul Islam[8], Lloyd W. Sumner[4,8,9], Trupti Joshi[3,4,10], and Cheryl S. Rosenfeld[1,11,12,13]

Departments of:

[1]Pathobiology and Integrative Biomedical Sciences. University of Missouri, Columbia, Missouri, 65211, USA

[2]Biological Sciences, University of Missouri, Columbia, Missouri, 65211, USA

[3]Department of Biomedical Sciences, Joan C. Edwards School of Medicine, Marshall University, Huntington, West Virginia, USA

[4]Christopher S. Bond Life Science Center, University of Missouri, Columbia, Missouri, 65211, USA

[5]Animal Sciences, University of Missouri, Columbia, Missouri, 65211, USA

[6]Obstetrics, Gynecology, and Women's Health, University of Missouri, Columbia, Missouri, 65211, USA

[7]Genomics Technology Core Facility, University of Missouri, Columbia, Missouri, 65211, USA

[8]University of Missouri Metabolomics Center, University of Missouri, Columbia, Missouri 65211, USA

[9]Biochemistry, University of Missouri, Columbia, Missouri 65211.

[10] Department of Electrical Engineering and Computer Science, University of Missouri, Columbia, Missouri, 65201, USA

[11]MU Institute for Data Science and Informatics, University of Missouri, Columbia, Missouri, 65211, USA

[12]Department of Genetics Area Program, University of Missouri, Columbia, Missouri, 65211, USA

[13]Department of Thompson Center for Autism and Neurobehavioral Disorders, University of Missouri, Columbia, Missouri, 65211, USA

Grant Support: Studies were supported by 1R03HD114968-01A1 and a University of Missouri Research Council Grant to CSR. This work is supported by funding to TJ from Missouri Department of Health and Senior Services (MDHSS) - Contract #AOC23380006 and National Science Foundation (NSF) Cybersecurity Innovation OAC-2232889. Research reported in this publication was also supported by the National Institute Of General Medical Sciences of the National Institutes of Health under Award Number P20GM103434. The content is solely the responsibility of the authors and does not necessarily represent the official views of the National Institutes of Health. The research was also supported by unrestricted startup funds to TJ from Joan C. Edwards School of Medicine at Marshall University (MURC), Huntington, West Virginia, USA.

Short Title: Maternal Tryptophan Reduction and Placenta-Brain Axis

Keywords: Trophoblast; Pregnancy; Maternal Diet; Transcriptomics; Metabolomics; Serotonin, Nutrition

Correspondence: Joshitr@marshall.edu or rosenfeldc@missouri.edu

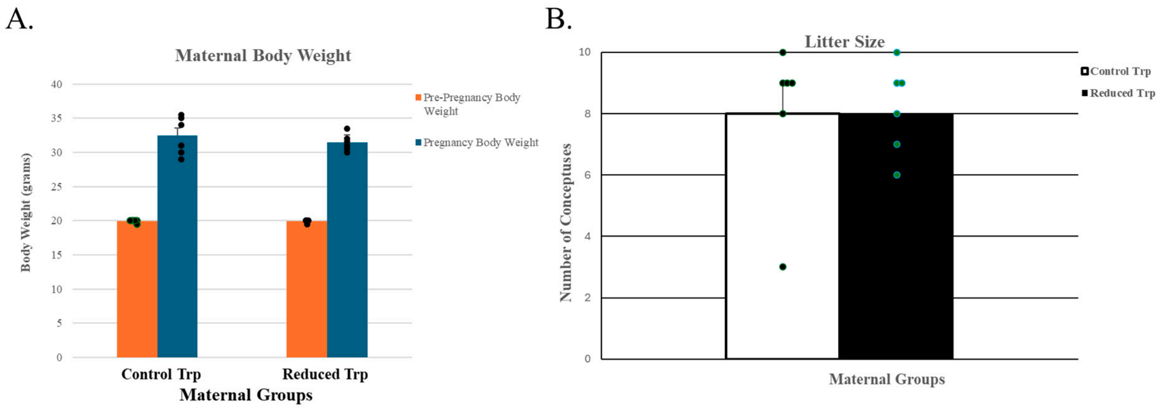

Figure S1. Comparison of pre and post-body weight and litter size in reduced and control Trp pregnant female mice. A) Body weight. B) No differences in litter size were noted between pregnant female mice on the reduced Trp diet compared to those on the control Trp diet (8.17 ± 0.64 vs. 8 ± 1.13 conceptuses, respectively, $p = 0.90$).

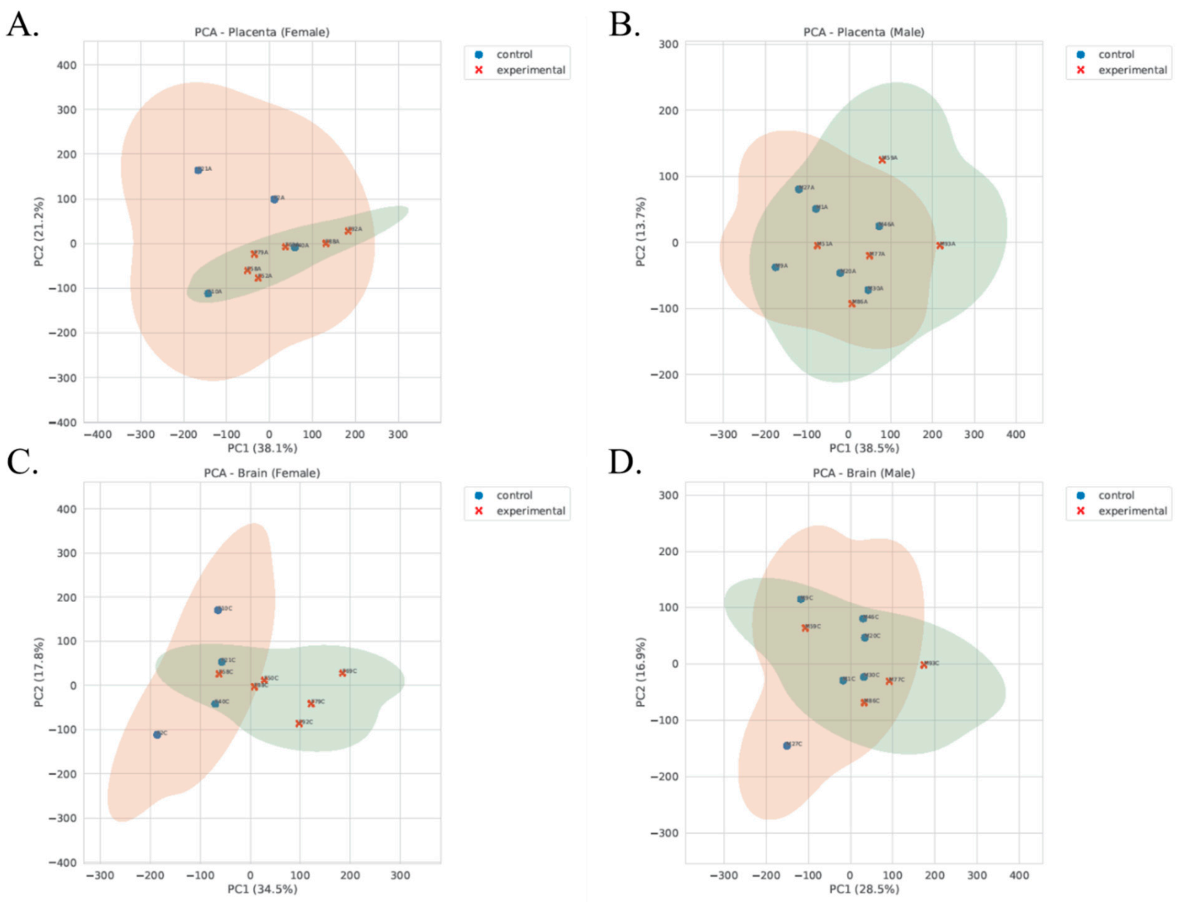

Figure S2. 2D PCA plot of RNAseq results from placenta and fetal brain samples from reduced Trp (0.1%) and control Trp (0.2%) diet. A) 2D PCA plot of RNAseq results for female placenta from conceptuses derived from reduced Trp (0.1%) vs. female placenta from conceptuses derived from control Trp (0.2%) dams. B) 2D PCA plot of RNAseq results for male placenta from conceptuses derived from reduced Trp (0.1%) vs. male placenta derived from conceptuses control Trp (0.2%) dams. C) 2D PCA plot of RNAseq results for female fetal brain from conceptuses derived from reduced Trp (0.1%) vs. female fetal brain from conceptuses derived from control Trp (0.2%) dams. D) 2D PCA plot of RNAseq results for male fetal brain from conceptuses derived from reduced Trp (0.1%) vs. male fetal brain from conceptuses derived from control Trp (0.2%) dams. PERMANOVA values for female and male placental comparisons were 0.05 and 0.23, respectively. PERMANOVA values for female and male fetal

brain comparisons were 0.04 and 0.26, respectively. Thus, there was significant separation for the female group comparisons but not for the male group comparisons.

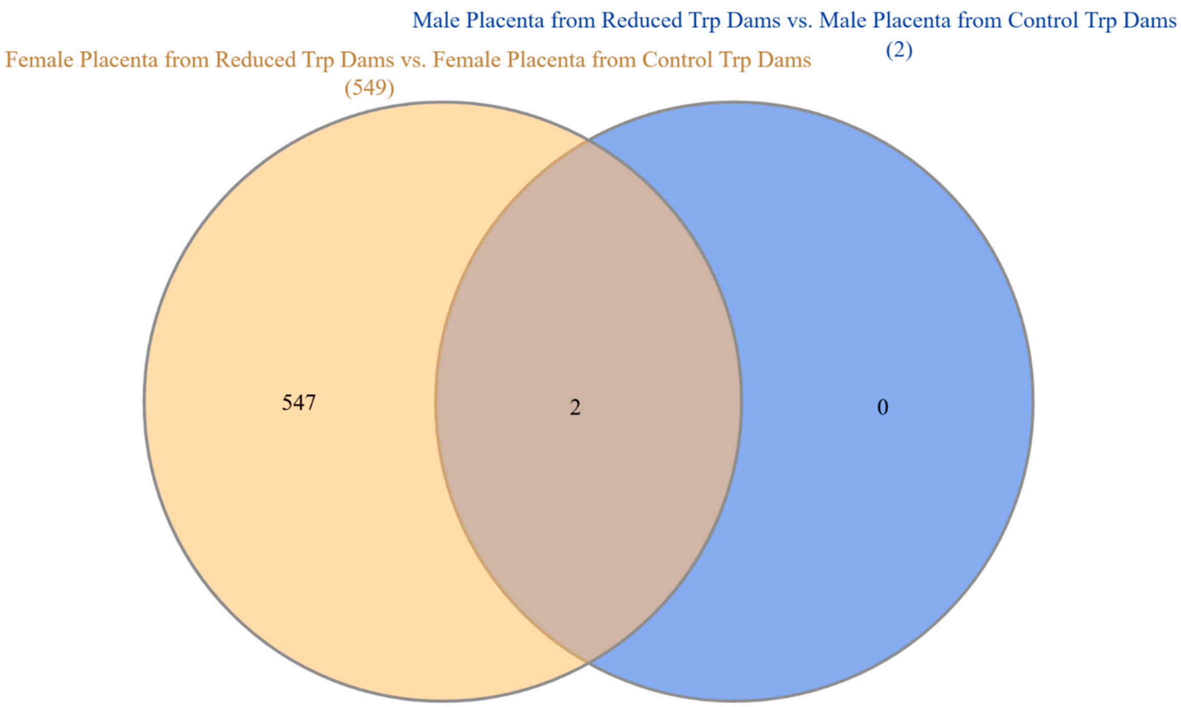

Figure S3. Venn diagram for overlap in transcripts when comparing differentially expressed genes in female placenta derived from reduced Trp vs. control Trp dams compared to differentially expressed genes in male placenta derived from reduced Trp vs. control Trp dams. Of the 549 known differentially expressed transcripts in female placenta compared to the two known differentially expressed genes in male placenta, both genes differentially expressed in the male (*Car6* and 1810073O08Rik*)* overlapped when comparing reduced Trp vs. Control Trp placental samples. List of genes that are solely differentially expressed in female placenta from reduced Trp vs. Control Trp dams in column A, and list of differentially expressed genes that overlap in female and male comparisons in column B.

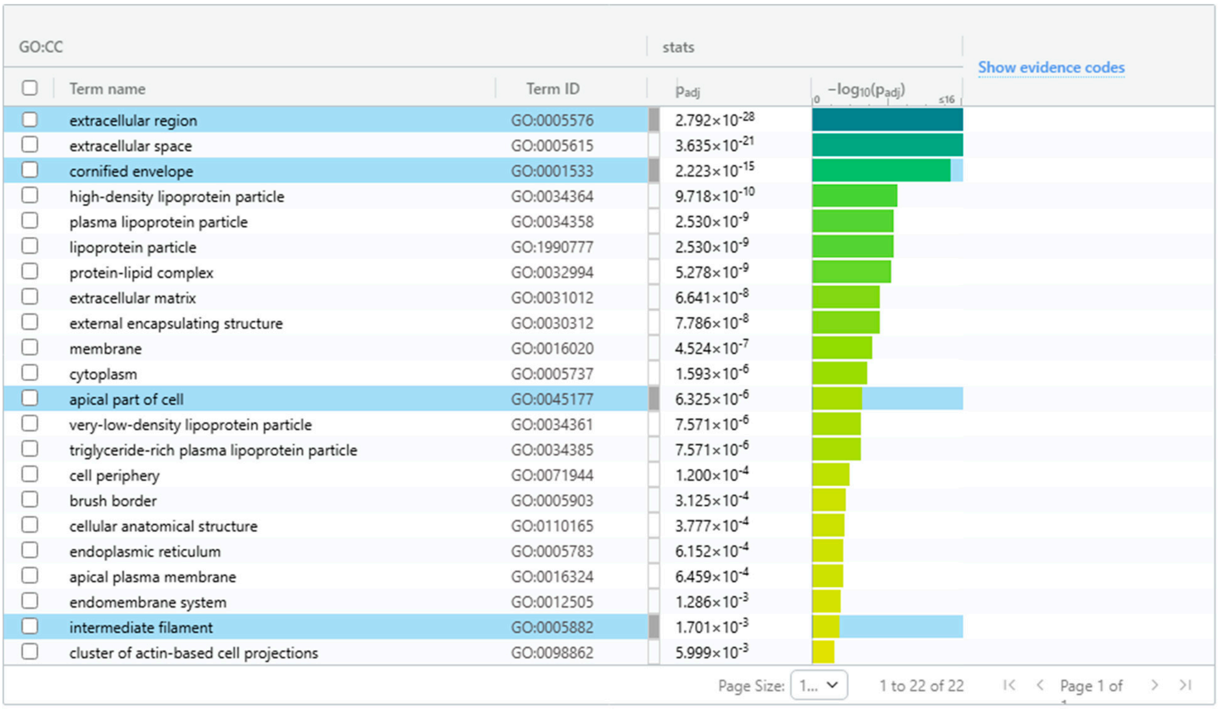

Term name	Term ID	p_{adj}
extracellular region	GO:0005576	2.792×10^{-28}
extracellular space	GO:0005615	3.635×10^{-21}
cornified envelope	GO:0001533	2.223×10^{-15}
high-density lipoprotein particle	GO:0034364	9.718×10^{-10}
plasma lipoprotein particle	GO:0034358	2.530×10^{-9}
lipoprotein particle	GO:1990777	2.530×10^{-9}
protein-lipid complex	GO:0032994	5.278×10^{-9}
extracellular matrix	GO:0031012	6.641×10^{-8}
external encapsulating structure	GO:0030312	7.786×10^{-8}
membrane	GO:0016020	4.524×10^{-7}
cytoplasm	GO:0005737	1.593×10^{-6}
apical part of cell	GO:0045177	6.325×10^{-6}
very-low-density lipoprotein particle	GO:0034361	7.571×10^{-6}
triglyceride-rich plasma lipoprotein particle	GO:0034385	7.571×10^{-6}
cell periphery	GO:0071944	1.200×10^{-4}
brush border	GO:0005903	3.125×10^{-4}
cellular anatomical structure	GO:0110165	3.777×10^{-4}
endoplasmic reticulum	GO:0005783	6.152×10^{-4}
apical plasma membrane	GO:0016324	6.459×10^{-4}
endomembrane system	GO:0012505	1.286×10^{-3}
intermediate filament	GO:0005882	1.701×10^{-3}
cluster of actin-based cell projections	GO:0098862	5.999×10^{-3}

Figure S4. GO CC pathways associated with DEG in female placenta from reduced Trp dams vs. control Trp dams. GO CC pathways associated with differentially expressed transcripts in female placenta include those associated with apical part of the cell, intermediate filament, and extracellular region. Other cellular components/pathways are shown in this figure.

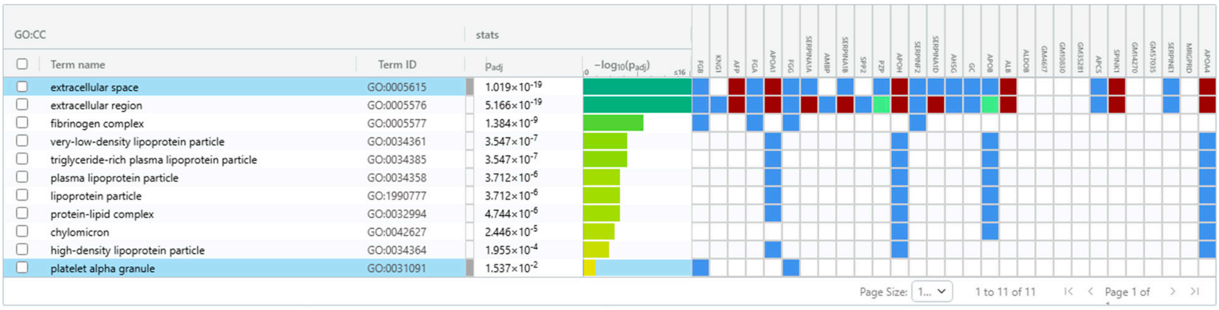

Figure S5. GO CC Pathways associated with DEG in female fetal brain from reduced Trp dams vs. control Trp dams. GO CC processes enriched for differentially expressed transcripts in female fetal brain comparison include extracellular space and platelet alpha granule. Other pathways are also shown in this figure.

See enclosed PDF file.

Figure S6. Correlation analyses of differentially expressed genes in female placenta from reduced Trp dams vs. control Trp dams and concentrations of Trp, 5-HT, and 5-HIAA in this organ. This diagram included the full list of DEGs in female placenta and their association with these metabolites. A key to the spectrum of blue to red colors is below the figure. Those correlations that are significant at a p value < 0.05 are designated with a red box.

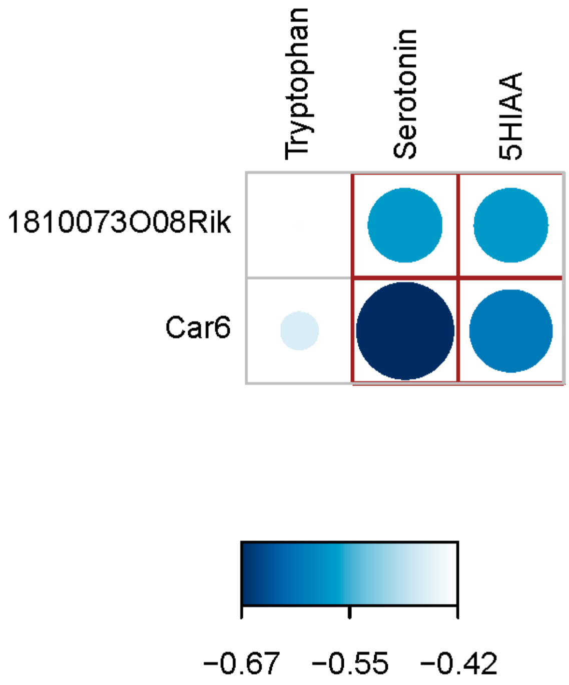

Figure S7. Correlation analyses of differentially expressed genes in male placenta from reduced Trp dams vs. control Trp dams and concentrations of Trp, 5-HT, and 5-HIAA in this organ. This diagram included the full list of DEGs in female placenta and their association with these metabolites. A key to the spectrum of blue to red colors is below the figure. Those correlations that are significant at a p value < 0.05 are designated with a red box.

Table S1. Dietary composition of reduced Trp (0.1%) diet that was provided prior to conception and during pregnancy to the mice in this study.

TD.190563

0.1% Trp Diet (O)

Formula	g/Kg
L-Alanine	3.5
L-Arginine	10.0
L-Asparagine	6.0
L-Aspartic Acid	3.5
L-Cystine	3.5
L-Glutamic Acid	40.0
Glycine	23.3
L-Histidine	3.3
L-Isoleucine	8.2
L-Leucine	11.1
L-Lysine HCl	18.0
L-Methionine	8.2
L-Phenylalanine	7.5
L-Proline	3.5
L-Serine	3.5
L-Threonine	8.2
L-Tryptophan	1.0
L-Tyrosine	5.0
L-Valine	8.2
Sucrose	343.98
Corn Starch	150.0
Maltodextrin	150.0
Soybean Oil	80.0
Cellulose	30.0
Mineral Mix, AIN-93M-MX (94049)	35.0
Calcium Phosphate, monobasic, monohydrate	8.2
Sodium Bicarbonate	5.0
Vitamin Mix, AIN-93-VX (94047)	19.5
Choline Bitartrate	2.7
TBHQ, antioxidant	0.02
Orange Food Color	0.1

L- **Footnote**

L- Modified from TD.99366, with 0.1% Trp for low level, and to use free base versions of Arg and
L- His to reduce Cl. 0.5% sodium bicarbonate added for buffer. Orange food coloring is added for
L- visual distinction.
L-
L-

analyze. answer. advance.

Key Features

- **Amino Acid Defined Diet**
- **Tryptophan - Low**
- **Color Coded Orange**

Selected Nutrient Information [1]

	% by weight	% kcal from
Protein [2]	15.3	15.6
CHO	64.8	66.1
Fat	8.0	18.4
Kcal/g	3.9	

[1] Calculated values

[2] Protein based on N x 6.25

Teklad Diets are designed & manufactured for research purposes only.

Key Planning Information

- *Products are made fresh to order*
- Store product at 4°C or lower
- Use within 6 months (applicable to most diets)
- Box labeled with product name, manufacturing date, and lot number
- Replace diet at minimum once per week
  More frequent replacement may be advised
- Lead time:
  · 2 weeks non-irradiated
  · 4 weeks irradiated

Product Specific Information

- 1/2" Pellet or Powder (free flowing)
- Minimum order 3 Kg
- Irradiation available upon request

Options (Fees Will Apply)

- Rush order (pending availability)
- Irradiation (see Product Specific Information)
- Vacuum packaging (1 and 2 Kg)

Place Your Order (USA & Canada)

Please Choose One

- **www.inotivco.com/teklad-orders**
- **tekladorders@inotivco.com**
- **800.483.5523**

Price Quotes | Order Status

- **teklad@inotivco.com**

Collaborate with a Nutritionist

- **askanutritionist@inotivco.com**

Table S2. Dietary composition of control Trp (0.2%) diet that was provided prior to conception and during pregnancy to the mice in this study.

TD.190359

0.2% Trp Diet (Control, G)

Formula	g/Kg
L-Alanine	3.5
L-Arginine	10.0
L-Asparagine	6.0
L-Aspartic Acid	3.5
L-Cystine	3.5
L-Glutamic Acid	40.0
Glycine	23.3
L-Histidine	3.3
L-Isoleucine	8.2
L-Leucine	11.1
L-Lysine HCl	18.0
L-Methionine	8.2
L-Phenylalanine	7.5
L-Proline	3.5
L-Serine	3.5
L-Threonine	8.2
L-Tryptophan	2.0
L-Tyrosine	5.0
L-Valine	8.2
Sucrose	342.98
Corn Starch	150.0
Maltodextrin	150.0
Soybean Oil	80.0
Cellulose	30.0
Mineral Mix, AIN-93M-MX (94049)	35.0
Calcium Phosphate, monobasic, monohydrate	8.2
Sodium Bicarbonate	5.0
Vitamin Mix, AIN-93-VX (94047)	19.5
Choline Bitartrate	2.7
TBHQ, antioxidant	0.02
Green Food Color	0.1

L- **Footnote**

L- Modified from TD.99366, with 0.2% Trp for control level, and to use free base versions of Arg
L- and His to reduce Cl. 0.5% sodium bicarbonate added for buffer. Green food coloring is added
L- for visual distinction.

L-

L-

inotiv
analyze. answer. advance.

Key Features

- **Amino Acid Defined Diet**
- **Tryptophan Control**
- **Color Coded Green**

Selected Nutrient Information [1]

	% by weight	% kcal from
Protein [2]	15.4	15.7
CHO	64.7	66.0
Fat	8.0	18.4
Kcal/g	3.9	

[1] Calculated values

[2] Protein based on N x 6.25

Teklad Diets are designed & manufactured for research purposes only.

Key Planning Information

- *Products are made fresh to order*
- Store product at 4°C or lower
- Use within 6 months (applicable to most diets)
- Box labeled with product name, manufacturing date, and lot number
- Replace diet at minimum once per week
  More frequent replacement may be advised
- Lead time:
  - 2 weeks non-irradiated
  - 4 weeks irradiated

ISO 9001 SGS

Product Specific Information

- 1/2" Pellet or Powder (free flowing)
- Minimum order 3 Kg
- Irradiation available upon request

Options (Fees Will Apply)

- Rush order (pending availability)
- Irradiation (see Product Specific Information)
- Vacuum packaging (1 and 2 Kg)

Place Your Order (USA & Canada)

Please Choose One

- **www.inotivco.com/teklad-orders**
- **tekladorders@inotivco.com**
- **800.483.5523**

Price Quotes | Order Status

- **teklad@inotivco.com**

Collaborate with a Nutritionist

- **askanutritionist@inotivco.com**

062724

Table S3. Number of raw reads, mapped reads, and % mapped reads from RNAseq results from placenta and fetal brain samples from pregnant female mice on the control Trp diet (0.2%) and reduced Trp (0.1%) diet.

**Sample ID**	**Maternal Treatment (Control or Low Trp Diet)**	**Sex**	**Organ**	**Raw Total Reads**	**Mapped Reads**	**% of Mapped Reads**
M1A	Control Trp	Male	Placenta	1.14E+08	1.12E+08	94.7
M9A	Control Trp	Male	Placenta	1.08E+08	1.06E+08	95.4
M20A	Control Trp	Male	Placenta	1.29E+08	1.26E+08	92.6
M27A	Control Trp	Male	Placenta	1.14E+08	1.11E+08	94.4
M30A	Control Trp	Male	Placenta	1.35E+08	1.32E+08	94.4
M46A	Control Trp	Male	Placenta	1.51E+08	1.48E+08	96.5
F2A	Control Trp	Female	Placenta	1.36E+08	1.33E+08	94.8
F10A	Control Trp	Female	Placenta	80878344	79514095	95.4
F21A	Control Trp	Female	Placenta	1.39E+08	1.35E+08	93.2
F40A	Control Trp	Female	Placenta	1.35E+08	1.33E+08	96.7
M1C	Control Trp	Male	Fetal Brain	1.5E+08	1.48E+08	94.2
M9C	Control Trp	Male	Fetal Brain	1.28E+08	1.26E+08	95.5
M20C	Control Trp	Male	Fetal Brain	1.37E+08	1.34E+08	91.1
M27C	Control Trp	Male	Fetal Brain	1.01E+08	99305429	93.7
M30C	Control Trp	Male	Fetal Brain	1.2E+08	1.17E+08	94.1
M46C	Control Trp	Male	Fetal Brain	1.45E+08	1.43E+08	96.9
F2C	Control Trp	Female	Fetal Brain	84038966	82750675	95.2
F10C	Control Trp	Female	Fetal Brain	1.33E+08	1.3E+08	95.7
F21C	Control Trp	Female	Fetal Brain	1.21E+08	1.19E+08	93.5
F40C	Control Trp	Female	Fetal Brain	1.05E+08	1.03E+08	96.8
M51A	Reduced Trp	Male	Placenta	1.19E+08	1.17E+08	96.5
M59A	Reduced Trp	Male	Placenta	1.55E+08	1.52E+08	95.1
M77A	Reduced Trp	Male	Placenta	1.58E+08	1.55E+08	96.1
M86A	Reduced Trp	Male	Placenta	1.26E+08	1.24E+08	94.5
M93A	Reduced Trp	Male	Placenta	1.74E+08	1.71E+08	94.7
F52A	Reduced Trp	Female	Placenta	1.1E+08	1.08E+08	96.8
F58A	Reduced Trp	Female	Placenta	1.02E+08	99857598	95.8

F69A	Reduced Trp	Female	Placenta	1.38E+08	1.35E+08	96.1
F79A	Reduced Trp	Female	Placenta	1.11E+08	1.09E+08	95.6
F88A	Reduced Trp	Female	Placenta	1.61E+08	1.59E+08	95.3
F92A	Reduced Trp	Female	Placenta	1.55E+08	1.52E+08	94.1
M59C	Reduced Trp	Male	Fetal Brain	1.24E+08	1.23E+08	97.1
M77C	Reduced Trp	Male	Fetal Brain	1.47E+08	1.44E+08	94.8
M86C	Reduced Trp	Male	Fetal Brain	1.31E+08	1.29E+08	95.2
M93C	Reduced Trp	Male	Fetal Brain	1.43E+08	1.41E+08	94.7
F50C	Reduced Trp	Female	Fetal Brain	1.27E+08	1.25E+08	96.7
F58C	Reduced Trp	Female	Fetal Brain	1.19E+08	1.17E+08	94.6
F69C	Reduced Trp	Female	Fetal Brain	1.79E+08	1.76E+08	96.5
F79C	Reduced Trp	Female	Fetal Brain	1.44E+08	1.41E+08	95.8
F88C	Reduced Trp	Female	Fetal Brain	1.21E+08	1.19E+08	95.8
F92C	Reduced Trp	Female	Fetal Brain	1.28E+08	1.26E+08	94.7
**Average**				1.31E+08	1.28E+08	95.2

Table S4. Tryptophan (Trp), serotonin (5-HT), and 5-HIAA concentrations in maternal serum and uterine tissue. Concentrations are on ng/µL for maternal serum and ng/mg for uterine tissue.

	**Trp**	**5-HT**	**5-HIAA**
**Control Maternal Serum**	13.05 ± 2.45	1.62 ± 0.75	0.077 ± 0.02
**Low Trp Maternal Serum***	6.96 ± 2.83	0.803 ± 0.59	0.106 ± 0.08
**Control Uterine Tissue**	8.26 ± 1.81	0.001 ± 0.0004	0.22 ± 0.05
**Low Trp Uterine Tissue**	9.45 ± 2.96	0.001 ± 0.0005	0.24 ± 0.07

*Two of the serum samples for the low Trp female mice were insufficient to measure these metabolites. Thus, for this group, only four replicates were tested. For all other groups, six replicates were tested.

Table S5. Tryptophan (Trp), serotonin (5-HT), and 5-HIAA concentrations in placenta and fetal brain samples from female . Concentrations are in ng/mg tissue.

	**Trp**	**5-HT**	**5-HIAA**
**Female placenta from Control dams**	23.8 ± 1.93	0.21 ± 0.04	0.44 ± 0.08
**Female placenta from dams on a low Trp diet**	16.43 ± 0.66[a]	0.07 ± 0.02[c]	0.24 ± 0.07[b]
**Male placenta from control dams**	23.5 ± 2.17	0.30 ± 0.07	0.54 ± 0.10
**Male placenta from dams on a low Trp diet**	15.86 ± 0.60[c]	0.08 ± 0.03[P = 0.01]	0.20 ± 0.04[d]
**Female fetal brain from Control dams**	51.94 ± 3.37	0.04 ± 0.01	0.31 ± 0.07
**Female fetal brain from dams on a low Trp diet**	25.04 ± 2.01[e]	0.01 ± 0.007[b]	0.17 ± 0.04[f]
**Male fetal brain from control dams**	45.6 ± 3.41	0.072 ± 0.03	0.29 ± 0.05
**Male fetal brain from dams on a low Trp diet**	24.2 ±1.90[g]	0.0075 ± 0.03[h]	0.12 ± 0.04[i]

[a]p = 0.001; [b]p ≤ 0.05; [c]p = 0.007; [d]p = 0.007; [e]p = 0.00005; [f]p = 0.06;[g]p = 0.0003; [h]p =0.08; [i]p=0.02
